# Immune-Centered Cross-Talk Between Cancer Cells and the Tumor Microenvironment—Implications for Therapy

**DOI:** 10.3390/cancers18030344

**Published:** 2026-01-23

**Authors:** Eliza Turlej, Aleksandra Domaradzka, Rostyslav Koksharov, Agnieszka Gizak

**Affiliations:** Department of Molecular Physiology and Neurobiology, University of Wrocław, ul. Sienkiewicza 21, 50-335 Wrocław, Poland; aleksandra.domaradzka@uwr.edu.pl (A.D.); 34623@uwr.edu.pl (R.K.)

**Keywords:** cancers, tumor microenvironment, immune cells, anti-cancer therapies

## Abstract

The tumor microenvironment (TME), composed of various immune and non-immune cells, as well as cancer stem cells, plays a critical role not only in promoting cancer cell proliferation and metastasis but also in modulating therapeutic response. A wide range of therapeutic strategies targeting the TME are currently employed in cancer treatment, including standard chemotherapy, radiotherapy, immunotherapy, anti-angiogenic therapies, agents targeting cancer-associated fibroblasts (CAFs), oncolytic viruses (OVs), cold atmospheric plasma therapy, and nanovaccines. This review provides a comprehensive overview of the influence of the TME on cancer sensitivity to these therapies across all types of solid tumors.

## Simple Summary

Cancer develops in a complex environment composed of many different cells that can either support or hinder treatment. Modern therapies, including immune-based approaches, increasingly target not only the cancer cells themselves but also the surrounding cells that support tumor growth. In this review, we describe how different treatment methods influence the tumor environment and how this knowledge can lead to more effective and personalized therapies. We also present new strategies that use modified immune cells, such as engineered macrophages, to improve the body’s ability to recognize and destroy cancer. Understanding how therapies interact with the tumor environment can help scientists design better therapies and improve outcomes for patients in the future.

## 1. Introduction

Recent studies have shown that successful clinical treatment outcomes in cancer patients depend not only on the complete elimination of cancer cells without affecting healthy ones, but also on the inhibition of pro-cancer effects of surrounding cells. The surrounding cancer environment mainly consists of a wide variety of immune cells, including lymphocytes, monocytes/macrophages, dendritic cells (DCs), myeloid-derived suppressor cells (MDSCs), granulocytes, cancer stem cells (CSCs), fibroblasts, endothelial cells, and mesenchymal cells [1,2]. The characteristics and roles of these surrounding cells in cancer development have been summarized by our group in a previous paper [3]. Here, we focus on the role of targeted anti-cancer therapies directed towards the tumor microenvironment (TME).

The main aim of targeted therapies in the context of the TME and tumor organismal environment (TOE) is to reshape their composition in order to eliminate survival signals for cancer cells. According to modern concepts, the TOE represents microenvironments distant from the primary cancer lesion that affect its development and include exosomes and microbiota [4]. Unfortunately, the TME is a dynamic environment that undergoes numerous changes throughout the entire course of cancer treatment, which limits the effectiveness of therapy. Considering the heterogeneity of cancers and the additional factors influencing therapeutic responses, it is difficult to select an appropriate treatment. Nevertheless, immunotherapy, anti-angiogenic drugs, and drugs targeting CAFs are among the most successful approaches. In addition, OVs, CAP, and nanovaccines are also being explored.

In the following sections, we provide an overview of the impact of various anti-cancer therapies on the cellular components of the TME.

## 2. Chemotherapy Directed Against TME-Derived Cells

Among anti-cancer treatments, chemotherapy remains a leading approach and is often combined with surgery or radiotherapy, depending on tumor stage. Chemical agents affecting the TME include small-molecule inhibitors, peptides, antibodies, nanoparticles, and bifunctional systems combining therapeutic agents with imaging contrast particles or radioactive/chemoactive moieties. Chemotherapy is particularly effective in metastatic cancers; however, it often fails to completely eradicate cancer cells, which can rapidly recur after treatment cessation. The limited efficacy is largely attributed to CSCs present in the bloodstream. A major drawback of chemotherapy is its non-specific cytotoxicity, which inhibits proliferation not only of malignant but also rapidly dividing healthy cells such as intestinal epithelium, hair follicles, and bone marrow stem cells (BMSCs). Chemotherapeutic agents act through several mechanisms, including activation of intrinsic and extrinsic apoptotic pathways via Fas/FasL (Fas ligand) interaction, cytochrome C release, and p53-mediated cell cycle arrest through kinase inhibition. They also modulate autophagy via PI3K/mTOR (phosphatidylinositol 3-kinase/mammalian target of rapamycin) and MAPK (mitogen-activated protein kinase) pathways, alkylate DNA, inhibit DNA/RNA synthesis (particularly during the S phase), disrupt microtubule formation, induce DNA damage through reactive oxygen species (ROS), and inhibit topoisomerases [5].

Chemotherapy can also induce accelerated senescence in cancer cells, leading to inflammatory gene expression and chronic TME inflammation. Conversely, some chemotherapeutic agents may alleviate inflammation—for instance, low doses of cyclophosphamide can stimulate the secretion of inflammatory mediators (GM-CSF—granulocyte-macrophage stem cell factor, IL-1β, IL-5, IL-10, IFNγ—interferon gamma, TNFα—tumor necrosis factor alpha). Cyclophosphamide also suppresses immune responses by increasing MDSC recruitment and inducing ROS and reactive nitrogen species (RNS) production in the TME. Taxanes affect TNFα secretion, a key inflammatory factor in breast and ovarian cancers. Paclitaxel, used in breast cancer therapy, activates toll-like receptor 4 (TLR-4), promoting angiogenesis and metastasis, while stabilizing microtubules and disrupting mitosis. Doxorubicin (DOX), an anthracycline, inhibits topoisomerase II, damages intestinal epithelium, and increases IL-1β secretion through inflammasome activation. Cisplatin, a platinum-based drug, induces ROS generation, while oxaliplatin reduces programmed death ligand-1 (PD-L1) expression on DCs and enhances immune cell infiltration in murine colorectal cancer. Similarly, etoposide and 5-fluorouracil (5-FU) trigger inflammation in the TME. In preclinical models of advanced gastric cancer, 5-FU facilitates antigen presentation and selectively eliminates MDSCs [6,7,8,9].

Adaptive immune cells play a central role in cancer elimination, yet chemotherapeutic drugs can disrupt anti-cancer immunity by depleting B cells. For example, DOX significantly reduces B cells while increasing cytotoxic T cells (CTLs) and natural killer (NK) cells in breast cancer patients. Gemcitabine produces similar effects, decreasing B cells and antigen-specific IgG responses while enhancing T-cell activation. Although high doses of chemotherapy impair lymphocyte function, low doses may enhance immune responses by reducing MDSC and regulatory T cells (Treg) activity [10,11].

Conventional chemotherapeutic agents also show promise in TME-targeting combination therapies. Drugs such as aspirin, celecoxib, β-adrenergic antagonists, metformin, and statins—commonly used for cardiovascular diseases—exhibit TME-targeting properties and are under clinical evaluation alone or with standard chemotherapy. Drug repurposing offers major advantages: reduced development time and cost, lower clinical trial risk due to established safety, and faster clinical implementation once anti-cancer efficacy is confirmed [4].

Aspirin, due to its anti-platelet effect, exerts anti-cancer activity on the TME and can be used with PD-L1 blockade agents in breast cancers (BCs). Its ability to induce macrophage polarization toward the M1 phenotype, enhance macrophage-mediated clearance of cancer cell debris, and modulate Treg and MDSC function makes it attractive for combination therapy—for instance, in cervical and uterine cancers treated with vitamin D, cyclophosphamide, lansoprazole, pembrolizumab, and radiotherapy [4,12].

Aspirin modulates cancer metabolism by limiting glutaminolysis and enhancing glucose utilization within the TCA (tricarboxylic acid cycle), thereby sensitizing cancer cells to glutaminase 1 (GLS1) inhibition. In parallel, aspirin reduces pro-inflammatory cytokines, including IL-6, IL-1β, and monocyte chemoattractant protein-1 (MCP-1), attenuating the inflammatory milieu that supports tumor progression. Aspirin also interferes with TME interactions by disrupting cross-talk between cancer cells and macrophages or adipocytes, leading to decreased production of pro-angiogenic and immunosuppressive factors and promoting macrophage polarization toward the M1 phenotype. Additionally, aspirin suppresses desmoplastic features and stem-like traits of cancer cells, inhibits matrix metalloproteinase (MMP) activity, and limits extracellular matrix remodeling, collectively reducing tumor invasion and migration. These effects are mediated through modulation of multiple oncogenic signaling pathways, including nuclear factor kappa β (NF-κB), mTOR, PI3K/AKT, and Wnt/β-catenin, influencing cancer cell proliferation and apotosis [13,14,15].

Celecoxib, known as a cyclooxygenase-2 (COX-2) inhibitor, facilitates immune responses and inhibits cancer progression. When combined with neoadjuvant checkpoint inhibitors (CKIs), it may benefit colon cancer patients, as neoadjuvant CKIs enhance tumor-infiltrating lymphocyte (TIL) activation, improving clinical outcomes [16]. Celecoxib reduces the expression of pro-inflammatory and pro-tumorigenic cytokines, including IL-2, IL-4, IL-17A, and platelet-derived growth factor (PDGF), as well as mediators involved in macrophage and neutrophil recruitment within the tumor microenvironment. By attenuating immunosuppressive signaling, these effects may enhance the efficacy of immunotherapies. In parallel, celecoxib decreases the number of activated (alpha anti smooth muscle) α-SMA^+^ CAFs and limits myeloid cell infiltration, resulting in reduced cytokine-driven proliferation and migration. Inhibition of COX-2/prostaglandin E2 (PGE_2_) signaling further suppresses MMPs activity and the production of pro-angiogenic factors such as vascular endothelial growth factor (VEGF), thereby restricting extracellular matrix (ECM) remodeling and tumor invasion [17,18].

β-adrenergic antagonists block signaling pathways involved in cancer progression, angiogenesis, and metastasis. These drugs enhance MDSC immunosuppression and promote T-cell tumor infiltration, thereby increasing immunotherapy effectiveness. Metformin improves immunotherapy and targeted therapy outcomes in advanced pancreatic neuroendocrine cancer treated with everolimus, lanreotide, octreotide, or other somatostatin analogs. In breast cancer, metformin enhances the efficacy of anti-cytotoxic T-lymphocyte-associated protein 4 (anti-CTLA4) drugs and promotes T-cell infiltration, correlating with improved clinical results [19,20]. Metformin modulates the tumor microenvironment by reducing the secretion of cytokines that support M2 macrophage polarization while promoting M1-associated cytokine expression through adenosine monophosphate-activated protein kinase (AMPK)–NF-κB signaling, thereby fostering a more immunostimulatory environment. In addition, metformin attenuates extracellular matrix deposition by stromal cells, leading to reduced desmoplasia and potentially improved drug penetration. It also suppresses MMPs expression and mesenchymal markers while increasing epithelial markers such as E-cadherin, indicating reduction in ECM remodeling and inhibition of epithelial–mesenchymal transition (EMT) [21,22].

Drug repurposing also includes anti-fibrotic agents such as losartan and pirfenidone, which may be used for advanced solid tumors in pancreatic cancer (clinical trial NCT01821729—phase 2) and high-risk operable breast cancer (clinical trial NCT01101438—phase 3).

Mesenchymal stem cells (MSCs) can serve as delivery systems for chemotherapeutic agents such as DOX, gemcitabine, paclitaxel (PTX), and sorafenib. MSCs loaded with these drugs locally release them through passive diffusion within the tumor stroma, inducing cancer cell death [23].

## 3. Radiotherapy Directed Against TME-Derived Cells

Radiotherapy is applied in nearly 60% of newly diagnosed cancer patients, with its effectiveness evaluated by local tumor control. It serves as frontline therapy in many cancers, as an adjuvant treatment preventing recurrence after surgery, or as palliative care for advanced-stage disease [24].

Radiotherapy employs high-energy radiation to eliminate cancer cells by inducing double- and single-strand DNA breaks, misrepairs, and chromosomal aberrations. Cancer cells are primarily destroyed through mitotic catastrophe, apoptosis, necrosis, autophagy, or replicative senescence. Radiation also alters the TME by promoting pro-oxidant and pro-inflammatory conditions, transforming it into an immunological niche that supports adaptive immune responses. Markers of activation include major histocompatibility complex (MHC) proteins, cell adhesion molecules, cytokines and their receptors, and damage-associated molecular patterns (DAMPs), which act as immune stimulators [24].

Radiotherapy stimulates adaptive immunity and often requires higher fraction doses—three times greater than the standard 2 Gy—to release sufficient neoantigens and immunostimulatory molecules. Its efficacy, however, is limited by TME-associated hypoxia and the activity of Tregs, MDSCs, M2 tumor-associated macrophages (TAM M2), and anti-inflammatory cytokines such as tumor growth factor β (TGF-β). Many tumors are infiltrated by CD11b^+^ myeloid cells that differentiate into M2 macrophages in hypoxic regions. Blocking the hypoxia inducible factor α (HIF-1α)/stromal-derived factor (SDF-1)/CXC motif chemokin receptor-4) CXCR4 or colony stimulating factor receptor 1 (CSF1/CSF1R) axes can inhibit this infiltration and sensitize tumors to radiation [25,26].

Functional M1 macrophages in the TME enhance T cell-dependent immune responses, although some are polarized into M2 macrophages. Radiotherapy also promotes leukocyte infiltration by modifying vascular structure, increasing intracellular adhesion molecule 1 (ICAM-1) and vascular cell adhesion molecule 1 (VCAM-1) expression, and stimulating cytokine secretion (IL-1β, TNFα, IFN type I and II). Moreover, radiation promotes neutrophil migration into tumors, where they release ROS to kill cancer cells. Tumors with pro-inflammatory profiles generally show better responses to radiotherapy [25,26].

Radiation directly activates macrophages and induces immune effects mediated by DCs and macrophages in the irradiated TME. It can also suppress non-irradiated tumors through the release of pro-inflammatory signals and the activation of CTLs, which migrate to both irradiated and distant metastatic sites [27].

Radiotherapy stimulates MDSCs and their migration into inflamed tissues by inducing CD5 expression—a classical MDSC activator with radioprotective properties. MDSCs may differentiate into mature granulocytes or macrophages (M1 or M2); post-radiation, M2-like cells release high levels of immunosuppressive cytokines (TGFβ and IL-10). Similarly, radiotherapy elevates Treg levels, which reduce therapeutic efficacy since Tregs are among the most radioresistant lymphocytes due to high Akt expression. Combining radiotherapy with immunotherapy targeting CTLA-4 and PD-1/PD-L1 axes enhances immune activation in preclinical models and has driven the expansion of clinical trials combining these modalities [28,29].

## 4. Immunotherapy Directed Against TME-Derived Cells

**Figure 1:**
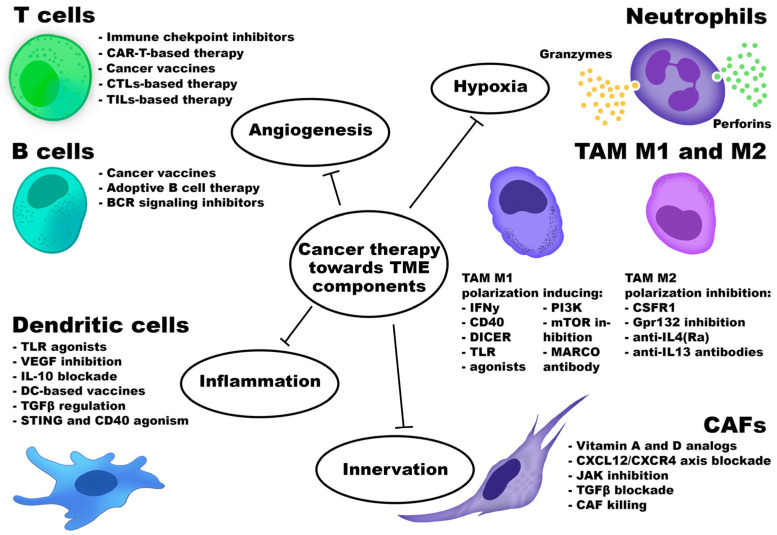
Schematic overview of therapeutic strategies targeting TME cells, discussed in this review. Detailed descriptions are provided in Section 4.1.1, Section 4.1.2, Section 4.1.3, Section 4.1.4 and Section 4.1.5.

Cancer immunotherapy was first introduced by William Coley, who stimulated the immune system in cancer patients through intratumoral injections of inactivated bacterial toxins [30]. Immune cells are among the most commonly cells targeted in cancer therapy. Figure 1 schematically illustrates the major therapeutic strategies directed against the TME component.

All immunotherapy approaches are generally classified as either active or passive. Active immunotherapy elicits a direct immune response against cancer-associated antigens expressed or overexpressed on the surface of tumor cells. These antigens may be proteins or carbohydrates unique to cancer cells. Passive immunotherapy, in contrast, uses monoclonal antibodies, lymphocytes, or cytokines to mediate anti-cancer effects. Overall, immunotherapy encompasses adoptive cell therapy (ACT), CKIs, targeted monoclonal antibodies, OV therapy, cancer vaccines, and cytokine-based treatments. Furthermore, the TME could be affected by small-molecule anti-cancer drugs, antibodies, peptides, vaccines, nanoparticles, and by gene therapy [31].

Small-molecule anti-cancer drugs primarily inhibit specific molecular targets essential for cancer cell proliferation and metabolism. These agents typically target proteins involved in cell signaling, metabolic regulation, or gene expression, and their binding to target molecules disrupts cellular functions that drive disease progression. To date, 57 small-molecule drugs have been approved for the treatment of solid tumors. Their main advantages include relatively simple and cost-effective synthesis and the possibility of oral administration, which improves patient convenience and is often associated with fewer adverse effects. However, their limitations include reduced efficacy against membrane-bound or extracellular targets, as well as challenges in achieving optimal dosing due to their broad effects on cellular metabolism [32].

Monoclonal antibody-based anti-cancer therapies offer several advantages, primarily due to their high specificity and affinity for defined antigens or proteins, which enhances treatment precision and limits off-target effects on healthy cells. These agents often exert rapid therapeutic activity and may induce sustained immunological responses. However, their clinical use is limited by high production costs, the need for relatively large doses to achieve therapeutic efficacy, and the risk of serious adverse effects. Such side effects are frequently associated with intrinsic immunogenicity, immunosuppressive effects on surrounding cells, or excessive activation of immune responses [33].

Peptide drugs are chemically synthesized therapeutic peptides, typically composed of 10–50 amino acids, obtained through genetic recombination. Compared with small-molecule anti-cancer drugs, peptide-based agents act at lower concentrations, exhibit improved safety profiles, and often achieve favorable clinical outcomes, while offering good tissue penetration and relatively simple, cost-effective synthesis. Their main limitations include physicochemical instability, short half-life, and poor oral bioavailability. These drawbacks can be mitigated through peptide modification, co-administration with protease inhibitors or permeability enhancers, and strategies that prolong circulation time, such as albumin binding [34].

Cancer vaccines offer the advantage of suppressing tumor progression by inducing durable antitumor immune memory, which may reduce the risk of recurrence. However, their efficacy is limited by interindividual variability and interference from the immune microenvironment [33].

**Figure 2:**
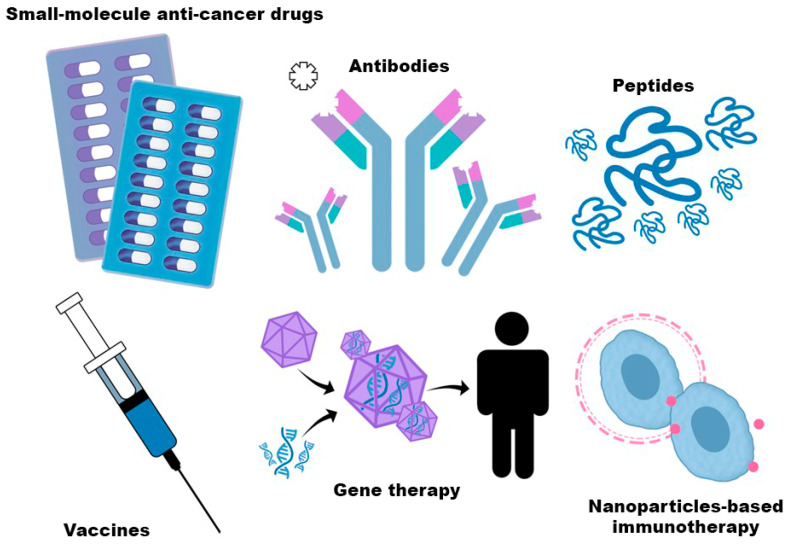
Molecules and strategies affected by the TME during immunotherapy. For a detailed description, please see the text above.

Gene therapy in cancer involves the delivery of DNA or RNA to modify gene expression or cellular functions, including gene replacement, inhibition, silencing, or supplementation. Its clinical application depends on tumor characteristics and patient-specific factors and is currently most often explored in combination with other treatments, particularly chemotherapy. Nevertheless, accumulating evidence suggests that gene therapy may evolve into a standalone option, owing to its potential for rapid and less toxic therapeutic effects [35]. Figure 2 presents the scheme of types of molecules and strategies affected by the TME during the immunotherapy.

The broad application of nanoparticles in cancer therapy is driven by their ability to improve drug bioavailability, stability, targeting, and therapeutic efficacy. Nanoparticles, typically defined as carriers smaller than 100 nm, can deliver small-molecule drugs, peptides, proteins, nucleic acids, or other bioactive compounds. Their key advantages include protection from degradation, prolonged circulation time, enhanced accumulation at tumor sites, and controlled drug release, which together optimize therapeutic outcomes. However, nanoparticles, particularly those based on inorganic materials, may exhibit biological toxicity related to their high surface area. In addition, their biodistribution, metabolism, and renal clearance critically influence both efficacy and safety [36].

### 4.1. Adoptive Cell Therapy

**Figure 3:**
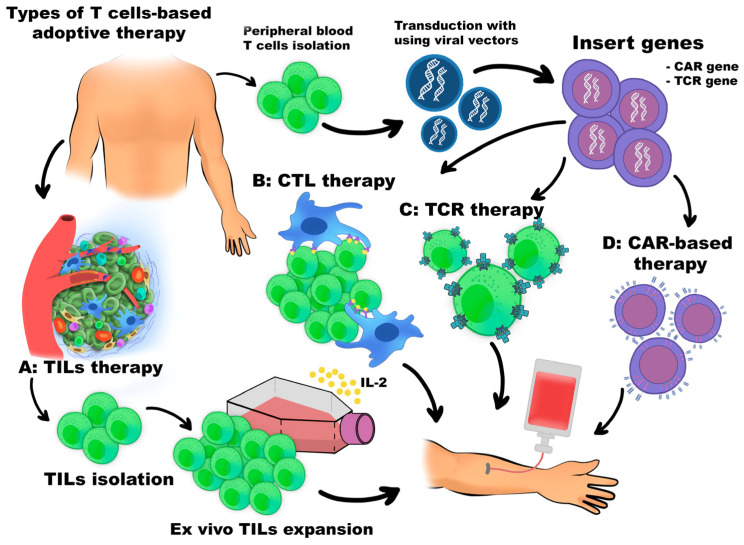
The most common types of adoptive cell therapy approaches used in cancer treatment. Detailed descriptions are provided in the corresponding sections of the manuscript. A: Tumor-infiltrating lymphocyte (TIL) therapy (Section 4.1.1), B: Cytotoxic T lymphocytes (CTLs) therapy, C: T-cell receptor-engineered T cell (TCR-T) therapy (Section 4.1.2), D: Chimeric antigen receptor T cells (CAR-T)-based therapy (Section 4.1.3).

ACT is a type of immunotherapy in which immune cells (mainly T cells) are given to a patient after primary chemotherapy. Types of ACT involve tumor-infiltrating therapy (TILs) and cytotoxic T cells-based therapy (CTLs-based therapy), T cell receptor (TCR) cells-based therapy and chimeric antigen receptor (CAR)-T cells, and CAR-NK cells or CAR-macrophages-based therapy—all described in detail in the below sections [37]. The most common types of ACT are schematically illustrated in Figure 3.

#### 4.1.1. TILs-Based Therapy

TILs therapy is based on the isolation of naturally infiltrating lymphocytes from tumor tissue, their in vitro expansion in the presence of cytokines (mainly IL-2), and reinfusion into patients. TILs consist predominantly of CTLs and Th cells; however, their antitumor activity within the TME is often impaired by immunosuppressive cells such as Tregs, MDSCs, and TAMs, as well as by inhibitory soluble factors. In addition, the low abundance of TILs in many solid tumors limits therapeutic efficacy [38].

Patient eligibility for TILs therapy depends on tumor type and stage, prior treatments, overall clinical status, and the feasibility of TIL isolation. Before TIL infusion, patients typically undergo non-myeloablative lymphodepletion with cyclophosphamide and fludarabine to reduce immunosuppressive cells and enhance TIL engraftment. IL-2 is administered to support TIL survival and expansion, although its use is associated with significant toxicity; IL-15 has therefore been proposed as a less toxic alternative that preferentially supports CTL activity. The effectiveness of TILs therapy is further limited by poor identification of neoantigen-specific T cells, T cell exhaustion induced by the TME, and the limited persistence of transferred cells in vivo. Emerging strategies, such as tumor-associated high endothelial venules (TA-HEVs), may improve T cell trafficking into tumors and serve as predictors of therapeutic response. Improved clinical outcomes have been observed when TILs therapy is combined with other treatments, particularly immune checkpoint inhibitors, which enhance antitumor immunity and help overcome resistance mechanisms. Additional combination approaches, including TILs with B-Raf proto-oncogene inhibitors (BRAF inhibitors), DC-based vaccines, or OVs, are currently under investigation to further improve therapeutic efficacy [39,40,41].

#### 4.1.2. Genetically Engineered T Cell Receptor Therapy

Modern bioengineering enables the insertion of specific antigen receptors into T cells, allowing antigen recognition in an MHC-dependent manner. TCRs, natural surface receptors on T cells, can be modified by altering their α or β chains [42].

TCRs undergo recombination in the thymus, generating a highly diverse TCR spectrum; however, the clonal frequency of antigen-specific T cells in peripheral blood is very low, which limits direct isolation of TCR-T cells. Therefore, enrichment strategies are required, including the use of TILs enriched in tumor-reactive clones, vaccination approaches, or selective in vitro expansion of peripheral blood T cells. The latter typically involves stimulation with autologous mature DCs loaded with target antigens, followed by genetic introduction of specific TCR genes using lentiviral or retroviral vectors. Modified T cells are then expanded in the presence of cytokines (IL-2, IL-15) and reinfused into patients [43].

TCR-T cells represent a promising approach for solid tumor treatment due to their high sensitivity to antigen levels and their ability to recognize both surface and intracellular antigens. They show efficient tumor infiltration and are generally associated with a lower incidence of severe adverse effects, such as cytokine release syndrome, compared with other adoptive cell therapies. TCR-T cells recognize intracellular proteins presented by MHC molecules more effectively than CAR-T cells (outlined in the section below) and use physiological signaling pathways, enhancing anti-cancer activity and reducing toxicity risk. However, compared to CAR-T cells, they exhibit lower avidity for target antigens and are restricted to specific MHC types, which limits the number of patients who can benefit from this therapy [44].

The main limitations of TCR-T cell-based therapy include the need for careful patient selection based on MHC molecules and the rapid development of resistance. Resistance often arises from MHC downregulation or heterogeneous expression, which impairs antigen presentation. Additional challenges include the lack of standardized protocols to define the optimal number of infused TCR-T cells and the risk of off-target toxicity, as TCR-T cells may also recognize low-level antigen expression in healthy tissues, exemplified by skin toxicity following melanoma antigen recognized by T cell 1 (MART-1)-directed therapy or inflammatory colitis in carcinoembryonic antigen (CEA)-targeted approaches. Another major limitation is the short persistence of transferred TCR-T cells, likely related to ex vivo expansion favoring late-memory phenotypes. This may be addressed by generating engineered T cells with a stem cell memory phenotype (Tscm) from naive precursors using IL-7, IL-15, or IL-21. Finally, the TME represents a substantial barrier to TCR-T therapy in solid tumors, as hypoxia and metabolic stress impair T cell proliferation and function. Limited oxygen availability, increased potassium levels, acidosis, and upregulation of immunosuppressive molecules such as CD39 collectively contribute to TCR-T cell dysfunction and reduced therapeutic efficacy [42,45,46].

#### 4.1.3. CAR-T Cells Therapy

Current research in T-cell-based therapies focuses mainly on CAR-T cells. This approach uses genetically modified T cells that target tumor cells overexpressing specific markers. CARs are engineered molecules designed to recognize a surface antigen on cancer cells and trigger T-cell activation through their intracellular domain [47].

The intracellular CAR domain includes the CD3ζ signaling domain and one or more co-stimulatory domains (CD28, 4-1BB, OX40) that enhance T-cell activation. The extracellular portion comprises a single-chain variable fragment (scFv) for antigen recognition, a hinge region that improves target accessibility, and a transmembrane domain that anchors the receptor to the T-cell membrane. Typically, CAR-T cells are autologous, though allogeneic sources are also possible. Their generation involves inserting a synthetic receptor into T cells, enabling them to recognize and kill target antigens before reinfusion into the patients [48].

**Figure 4:**
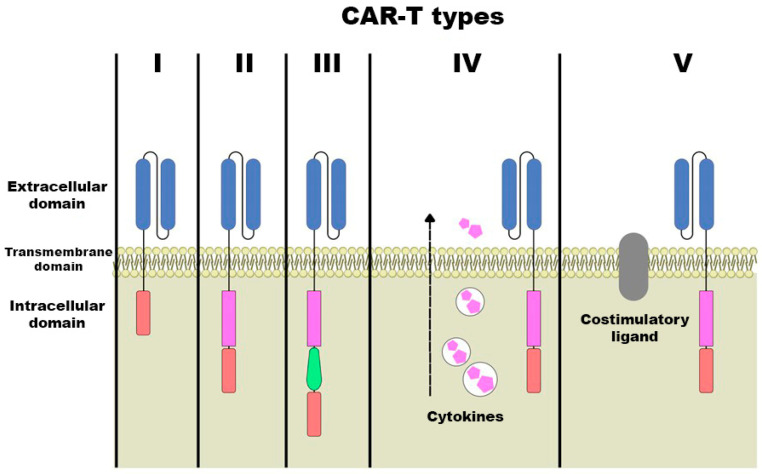
CAR-T cells generation. For a detailed description, please see the text. I—First generation of CAR-T cells, II—second generation of CAR-T cells, III—third generation of CAR-T cells, IV—fourth generation of CAR-T cells.

Five generations of CARs have been developed, differing in their intracellular co-stimulatory domains, which affect therapeutic efficacy. The first generation includes an extracellular antigen-recognition domain and a CD3ζ intracellular domain but lacks co-stimulatory signals, which limits activity of these CARs. The second generation introduces one co-stimulatory receptor domain, enhancing proliferation and cytokine release. The third generation adds two co-stimulatory molecules, further improving cytotoxicity. Later generations incorporate additional molecular components, including IL-related or suppressor genes, while the latest generation integrates three co-stimulatory domains and anti-PD-L1 scFv secretion directed against B-cell maturation antigen (BCMA), increasing antitumor efficacy and reducing T-cell exhaustion [49]. An overview of the five generations of CARs is presented in Figure 4.

New variants, such as nanobodies and single-domain antibodies (VHH), offer alternatives to scFvs. Notably, VHH-based CD19-redirected CAR-T cells demonstrate comparable proliferation, cytotoxicity, and immune responses [49].

To date, this therapy has been approved by the Food and Drug Administration (FDA) for leukemias—acute lymphoblastic leukemia (ALL)—and lymphomas, including large B-cell lymphoma (LBCL), follicular lymphoma (FL), mantle cell lymphoma (MCL), marginal zone lymphoma (MZL), and myeloma multiplex (MM) [50,51,52,53,54,55].

Expanding CAR-T therapy to solid tumors remains a key goal, although results so far have been modest—mainly due to limited tumor infiltration after intravenous administration, short CAR-T cell persistence, and the immunosuppressive TME. Promising targets for future applications include epithelial growth factor receptor (EGFR), human epidermal growth factor receptor 2 (HER-2), CEA, mesothelin (MSLN), epithelial cell adhesion molecule (EpCAM), and disialoganglioside (GD2). Most clinical studies have focused on glioblastoma, pleural, central nervous system, sarcoma, neuroblastoma, gastrointestinal, liver, and renal cancers. Breast and prostate cancers also appear promising due to overexpression of specific protein targets [56,57].

Immunosuppressive factors within the TME—particularly TAMs, Tregs, MDSCs, and tumor-associated fibroblasts (TAFs)—can inhibit CAR-T cell function. Cytokines such as TGF-β, IL-4, and IL-10 also contribute to this suppression, reducing therapy efficacy. A major complication of CAR-T therapy is immune effector cell-associated neurotoxicity syndrome (ICANS), a severe condition caused by massive cytokine release. CAR-T cell activity is influenced by inflammatory cytokines, including IL-2. Genetic engineering strategies now enable coupling T cells with synthetic cytokine circuits—e.g., cancer-specific synthetic Notch receptor (synNotch) linked to IL-2 production—which enhance CAR- or TCR-T-cell infiltration into the TME in pancreatic cancer and melanoma models [58].

Toxicity remains a major limitation of CAR-T cells application. Cytokine release syndrome (CRS) is a frequent inflammatory disorder caused by immune overactivation during CAR-T therapy, primarily involving IL-1, IL-6, IL-10, and IFNγ. Preventive strategies include using the IL-1 receptor antagonist Anakinra, generating CAR-T cells expressing IL-1R antagonists, and blocking IL-6R or GM-CSF, all of which have reduced CRS incidence. Additional barriers to CAR-T efficacy in solid tumors include the physical tumor barrier, difficulties identifying tumor-specific antigens, and limited trafficking or infiltration of CAR-T cells. Their function is further inhibited by hypoxia, acidic pH, nutrient deprivation, oxidative stress, suppressive cytokines and chemokines, Tregs, MDSCs, tumor-associated neutrophils (TANs), TAMs, and upregulation of inhibitory receptors on T cells [59,60,61].

#### 4.1.4. CAR-NK Cells Therapy

NK cells exhibit direct cytotoxicity against tumor and virus-infected cells and can mediate antibody-dependent cell-mediated cytotoxicity (ADCC) via the interaction between the IgG Fc fragment and CD16. Compared with CAR-T cells, CAR-NK cells are considered safer, as they are not associated with graft-versus-host disease (GvHD), CRS, or neurotoxicity, and do not require autologous cells.

CAR-NK cells can be derived from various sources, including peripheral blood of healthy donors, umbilical cord blood (UCB), induced pluripotent stem cells (iPSCs), or the NK-92 cell line (originally isolated from a patient with non-Hodgkin lymphoma). Peripheral blood NK cells—approximately 90% of which display a CD56^dim^CD16^bright^ phenotype—show strong cytotoxic potential. Similarly, UCB and NK-92 cells are valuable sources for CAR-NK generation [62].

However, NK cells derived from umbilical cord blood are less mature and exhibit lower cytotoxicity due to decreased expression of CD16, killer-immunoglobulin like receptors (KIRs), granzyme B, and perforins. The standardization of CAR-NK therapy is further challenged by variability in NK cell yield depending on the source. One solution involves differentiating CD34^+^ hematopoietic stem cells into NK cells using cytokine and chemokine cocktails, yielding CD3^−^CD56^+^ cells capable of killing tumor cells. Similarly, iPSC-derived NK cells represent a promising alternative due to their unlimited proliferative capacity and stable CAR expression [63,64].

The NK-92 cell line offers another practical option, as it can be efficiently transduced and expanded in large quantities. These cells are well-characterized, exhibit consistent immune responses, and have demonstrated safety in clinical applications. So far, CAR-NK cell therapy has been tested in a limited number of clinical trials targeting mucin-1 (MUC1)-positive relapsed or refractory solid and metastatic tumors, including pancreatic, ovarian, endometrial, prostate, glioblastoma, non-small cell lung, hepatocellular, triple-negative breast cancer (TNBC), gastroesophageal, colorectal, and head and neck cancers [57].

Despite promising safety and efficacy, CAR-NK therapy faces limitations, primarily related to the short half-life of NK cells, necessitating repeated administrations to achieve durable effects. Effective tumor trafficking and persistence within the TME remain critical challenges. Chemokines such as IL-8, CXCL1, and CXCL2 are under investigation as agents that could enhance CAR-NK migration to tumor lesions [65].

**Figure 5:**
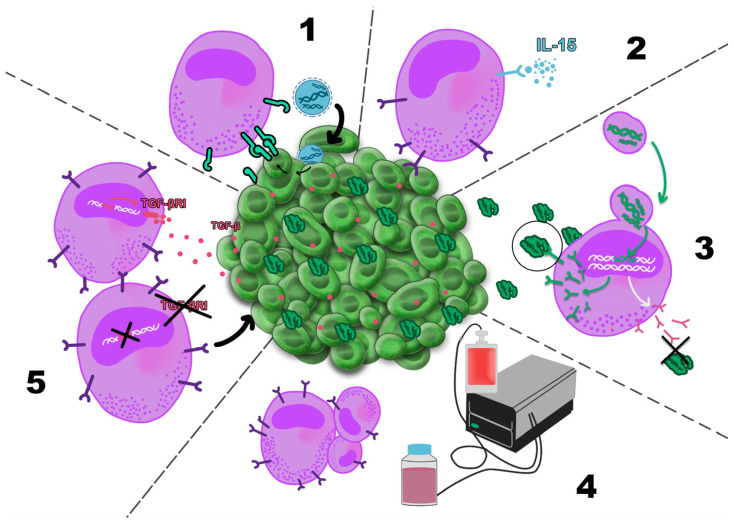
Immunotherapy models using CAR-NK cells. 1. Genetic engineering to modify chemokine receptor expression and improve tumor homing. 2. Cytokine supplementation to prolong NK cell persistence and activity. 3. Incorporation of co-stimulatory domains to improve receptor function. 4. Optimized transduction techniques to enhance CAR-NK activation and cytotoxicity. 5. Disruption of immunosuppressive signaling (TGFβ, adenosine or checkpoint pathways) responsible for enhanced resistance within the TME.

Physical barriers within the TME also restrict CAR-NK infiltration. For example, expression of fibroblast activation protein (FAP) in cancer-associated fibroblasts contributes to ECM remodeling, impeding immune cell access. Several strategies are being explored to optimize CAR-NK therapy in solid tumors. These include the following:

- Genetic engineering to modify chemokine receptor expression and improve tumor homing,
- Disruption of immunosuppressive signaling (TGFβ, adenosine, or checkpoint pathways), enhancing resistance within the TME,
- Cytokine supplementation to prolong NK cell persistence and activity,
- Addition of co-stimulatory domains to improve receptor function,
- Optimized transduction techniques to enhance CAR-NK activation and cytotoxicity as presented in Figure 5 [66].

Altogether, CAR-NK cells combine high antitumor potential with improved safety compared to CAR-T therapy, and ongoing optimization of their trafficking, persistence, and functional enhancement holds promise for future clinical applications in solid cancers.

#### 4.1.5. CAR-Macrophages Therapy

The success of CAR-T therapy and the strong potential of CAR-NK cells have stimulated growing interest in CAR macrophages (CAR-M) for cancer treatment. Like CAR-T and CAR-NK cells, CAR-M cells contain extracellular domains that recognize specific tumor antigens, transmembrane regions, and intracellular signaling domains. Current research on extracellular components focuses on well-established tumor targets such as CD19 and HER2, while studies on intracellular domains aim to enhance macrophage phagocytic capacity and functional activity [67].

The development of CAR-M offers new opportunities for treating solid tumors by modifying human macrophages with specific CARs, thereby improving their phagocytic activity and antigen presentation. CAR-M therapy involves transferring a defined CAR gene into macrophages, enabling them to recognize tumor-associated antigens, bind to cancer cells, and activate macrophage-mediated tumor destruction [68,69].

Various strategies have been explored to apply CAR-M in cancer therapy. CAR-147 macrophages were engineered to degrade the tumor extracellular matrix, enhance lymphocyte infiltration, and markedly increase intratumoral IL-12 and IFNγ levels. CAR-147 consists of a single-chain antibody fragment targeting human HER2, an IgG1 hinge, and the transmembrane and intracellular regions of mouse CD147. Intravenous administration of CAR-147 macrophages significantly inhibits tumor growth in mouse breast cancer models [67].

Chimeric Antigen Receptor-Phagocytes (CAR-Ps) were developed to direct macrophages to engulf specific targets. CAR-Ps express intracellular domains of Megf10 or IgY-Fc receptor (FcRγ), enhancing phagocytic activity toward target antigens. CAR-P_Megf10 specifically induces phagocytosis of the target ligand, with initiation triggered by tyrosine phosphorylation. However, CAR-P–target cell interactions alone do not induce full-cell engulfment. PI3K signaling plays a key role in promoting macrophage phagocytosis. Combining the PI3K p85 subunit with CAR-P–FcRγ to create a tandem receptor (CAR-P_tandem) enables whole-cell phagocytosis and significantly improves efficiency [70].

IPSCs engineered to express CARs and differentiate into macrophages and called CAR-iMacs have also been developed. In the absence of antigen, CAR-iMacs display features resembling M2 polarization. Upon encountering antigens such as leukemic or lymphomatous cells, they shift toward an inflammatory M1 phenotype. Stimulation by solid tumor cells further enhances CAR-iMac activity and phagocytosis. iPSC-derived macrophages may thus represent an important future component of cancer immunotherapy [71].

Compared with CAR-T cells, CAR-Ms offer several advantages. Macrophages infiltrate the TME more effectively, overcoming extracellular matrix barriers that often restrict T-cell penetration. CAR-Ms can also modulate the TAMs population by reducing their numbers and shifting the phenotype toward one that supports antitumor immunity. In addition to direct phagocytosis, CAR-Ms enhance antigen presentation and thereby promote stronger T-cell activation. Moreover, CAR-Ms display shorter persistence in circulation than CAR-T cells, which may help reduce systemic toxicity [72,73,74].

Despite these advantages, several limitations remain. Macrophages do not proliferate in vitro or after in vivo administration, restricting both therapeutic dosing and overall efficacy. Moreover, exogenously administered macrophages tend to traffic through the lungs and accumulate primarily in the liver, which may limit their effectiveness against certain solid tumors [75,76].

A promising strategy for improving CAR-M therapy is combining these cells with complementary treatments to further enhance phagocytic activity. This requires a deeper understanding of the regulatory mechanisms governing macrophage phagocytosis and antigen presentation, as well as the influence of the physical and structural properties of target cells. Such knowledge will be essential for the rational development and clinical application of CAR-M-based immunotherapies [77].

### 4.2. Immune Checkpoints Inhibitors (CKIs)

**Figure 6:**
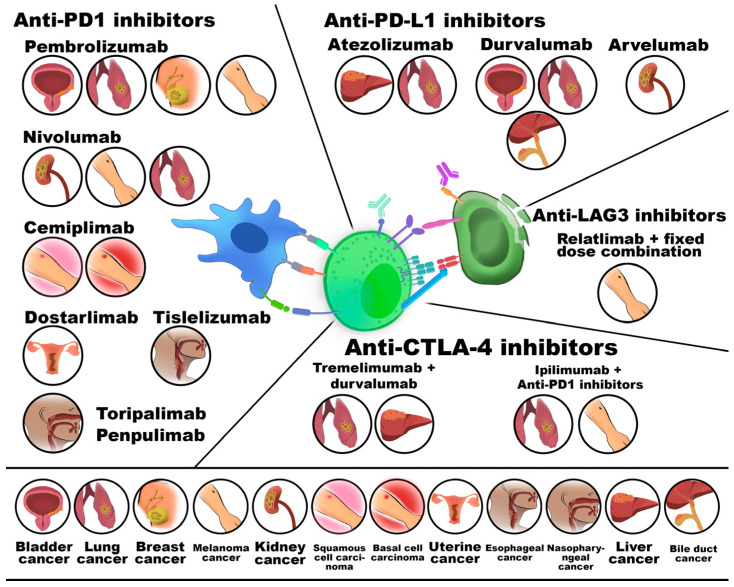
Schematic representation of CKI types used in various cancers.

Immune checkpoint molecules exert immunosuppressive functions and include PD-1, CTLA-4, T-cell immunoreceptor with Ig and ITIM domains (TIGIT), LAG-3 (lymphocyte activation gene 3), and MUC-3. These co-inhibitory receptors normally maintain immune tolerance and prevent excessive immune activation but can be therapeutically blocked by monoclonal antibodies. Since CKIs show bioactivity across various histological cancer types and provide stable clinical responses, they are used in metastatic and chemotherapy-resistant malignancies. In general, CKI–ligand interactions suppress T-cell function [78]. The heterogeneities of CKIs used in cancer treatment are presented in Figure 6.

CTLA-4 (CD152) is an inhibitory immune checkpoint that limits T-cell activation by competing with CD28 for binding to B7-1 (CD80) and B7-2 (CD86) on antigen-presenting cells (APCs). Its signaling suppresses naïve T-cell activation and contributes to immune tolerance, while its expression on Tregs further dampens immune responses within the TME. Blockade of CTLA-4 enhances antitumor immunity by promoting effector T-cell activity and reducing Treg-mediated suppression. Preclinical studies have demonstrated antitumor efficacy across multiple cancer types, and monoclonal antibodies such as ipilimumab and tremelimumab are clinically approved. However, CTLA-4 inhibition is frequently associated with immune-related adverse events, including gastrointestinal toxicity and hypophysitis [79,80,81,82,83,84].

PD-1 (CD279) is an inhibitory immune checkpoint expressed on activated T cells, B cells, and macrophages, playing a central role in immune tolerance. Its interaction with two ligands, PD-L1 (CD247) or PD-L2 (CD273), suppresses T-cell receptor signaling through recruitment of phosphatases and dephosphorylation of key TCR-associated molecules (ZAP-70 and CD3ζ), leading to reduced T-cell activation and function. PD-1 signaling additionally promotes ubiquitin-mediated degradation of signaling proteins, further reinforcing immune suppression. Therapeutic antibodies targeting the PD-1/PD-L1 axis, including pembrolizumab, nivolumab, cemiplimab, and others, restore antitumor immunity but are associated with immune-related adverse events such as thyroiditis, pneumonitis, and autoimmune diabetes. Combined blockade of PD-1 and CTLA-4 enhances efficacy but significantly increases toxicity [85,86,87].

LAG3 (CD223) is an inhibitory immune checkpoint expressed on multiple immune cell populations, including effector and regulatory T cells, NK cells, activated B cells, and plasmacytoid dendritic cells (pDCs). It is particularly enriched on exhausted T cells, where it suppresses proliferation and activation. By binding to human leukocyte antigen (HLA) class II molecules, LAG3 interferes with CD4-mediated T helper (Th) cell activation and promotes immune evasion in cancer. Therapeutic blockade of LAG3 enhances antitumor immune responses and is currently explored as monotherapy or in combination with PD-1/PD-L1 or CTLA-4 inhibitors, as well as through bispecific antibodies and fusion proteins [88,89].

T cell immunoglobulin and mucin-domain containing 3/hepatitis A virus cellular receptor 2 (TIM-3/HAVCR-2) plays a key role in immune regulation. The cytoplasmic tail of the protein includes tyrosine residues that facilitate interactions with the TCR complexes. TIM-3 binds several ligands: galectin—9 (Gal-9; induces Th1 apoptosis and regulates inflammation), carcinoembryonic antigen-related cell adhesion molecule 1 (CEACAM-1; controls immune tolerance and antitumor response), phosphatidylserine (PtdSer; induces apoptotic vesicles clearance), and high mobility group box 1 (HMGB1; a chromatin protein affecting innate immunity). Unbound TIM-3 associates with Bcl-2 antagonist of cell death 3 (BAT3), which recruits tyrosine kinases, promoting cytokine release and T cell proliferation [80,90].

TIM-3 regulates Th1/Th17 activity and supports phagocytosis and antigen cross-presentation by DCs. Its expression is controlled by T-bet, a Th1 transcription factor that enhances TIM-3 transcription during inflammation. Monoclonal antibodies targeting TIM-3 block its ligand interactions, restoring T cell activity and reducing exhaustion. These agents are used as monotherapy or combined with PD-1 inhibitors in the treatment of myeloid and solid tumors [91,92].

B7 homolog 3 protein (B7-H3, also known as CD276) is a type I transmembrane glycoprotein that can also exist in a soluble form. Functionally, B7-H3 can stimulate CD4^+^ and CD8^+^ T cell proliferation and cytotoxicity, yet in tumors, it primarily acts as an immune checkpoint inhibitor, facilitating immune evasion. Its overexpression on cancer cells correlates with the presence of immunosuppressive forkhead box P3 (FOXP3), elevated TGF-β1 and IL-10 levels, and poor prognosis. B7-H3 modulates the CCL2– C-C chemokine receptor type 2 (CCR2)–M2 axis, promoting monocyte recruitment and differentiation into tumor-associated macrophages. It also regulates neutrophil function: GM-CSF induces B7-H3 expression via the JAK–STAT3 pathway, enhancing neutrophil-mediated immunosuppression within the TME. Furthermore, B7-H3 inhibits NK cell activation by interfering with TLR-2 and TLR-4 signaling, and it suppresses CD4^+^ and CD8^+^ T cell activity, collectively contributing to tumor immune escape. Notably, its absence in resting T cells suggests that B7-H3 participates primarily in T cell expansion and activation processes [93].

TIGIT (also known as Vstm3, WUCAM, or VSIG9) belongs to the poliovirus receptor-like (PVR-like) protein family. It binds CD155 and CD112 on APCs, leading to suppression of T cell activation and NK cell cytotoxicity, thereby promoting immune tolerance. It is expressed on multiple immune cells, including CD4^+^, CD8^+^, and Tregs, as well as NK cells, in both mice and humans. Expression levels increase following immune cell activation. TIGIT interacts with several ligands—CD112, CD113, and CD155—members of the nectin-like (NECL) family that regulate immune responses. Among them, CD155 plays a central role; its overexpression in tumors such as melanoma, pancreatic, and lung cancers correlates with poor prognosis. High TIGIT expression is typically observed on T cells within the TME, while it remains low on naïve T cells. Dual blockade of TIGIT and PD-1 has shown synergistic effects, promoting CD8^+^ T cell expansion, enhancing TILs activity, and resulting in prolonged antitumor immunity and, in some models, complete tumor regression [94,95,96,97].

Another group of immune checkpoint inhibitors targets the CD47–SIRPα axis. CD47 is a transmembrane glycoprotein that protects healthy cells from immune attack. CD47 is widely expressed on normal cells, where its interaction with the signal regulatory protein (α SIRPα) receptor on macrophages suppresses phagocytosis and prevents immune-mediated clearance. Many cancer cells overexpress CD47 to evade immune surveillance, and elevated CD47 levels are associated with poor prognosis across multiple malignancies. Therapeutic blockade of the CD47–SIRPα pathway aims to restore macrophage-mediated tumor cell phagocytosis [98,99].

Among inhibitory receptors regulating innate immunity, Natural Killer Group 2 Member A (NKG2A) is expressed on NK cells and suppresses their cytotoxic activity through interaction with HLA-E. Cancer cells frequently exploit this pathway to escape NK cell-mediated killing, which correlates with reduced patient survival. While NKG2A blockade alone shows limited efficacy, its combination with PD-1/PD-L1 or EGFR inhibitors enhances antitumor immune responses [100].

VISTA (V-domain immunoglobulin suppressor of T cell activation) is a negative immune regulator mainly expressed in hematopoietic cells, modulating immune responses. It is highly present in neutrophils, monocytes, macrophages, dendritic cells, as well as on naïve CD4^+^ T cells and Tregs, controlling T cell activation and promoting immune suppression. Acting as a ligand, VISTA-Ig fusion strongly inhibits CD4^+^ and CD8^+^ T cell proliferation in both mouse and human models. Overall, VISTA functions as a co-inhibitory receptor on T cells, and agonistic antibodies targeting it effectively suppress CD4^+^ T cell activation. VISTA-interacting ligands, often overexpressed on cancer cells, weaken anti-cancer immune responses. The best-known anti-VISTA monoclonal antibody, W0180, can be used alone or with pembrolizumab, improving clinical outcomes in patients with head and neck cancers [101,102].

A novel immune checkpoint, poliovirus receptor-related immunoglobulin domain-containing (PVRIG/PVRL2), inhibits T cell activation, allowing cancer cells to evade immune detection. It is mainly expressed on CD8^+^ T cells and NK cells, but not on B cells, monocytes, or neutrophils, suggesting its specific regulatory role. PVRIG binds to PVLR2 (CD112/Nectin-2), which is often overexpressed in cancer cells, highlighting its role in tumor progression. Monoclonal antibodies against PVRIG slightly enhance NK cell cytotoxicity toward breast cancer cells in vitro. Combined anti-PVRIG and anti-TIGIT antibodies increase cytotoxicity against melanoma and pancreatic cancer cells [103,104].

Adenosine A2A Receptor/A2aR (ADORA2A), a G protein-coupled receptor, is expressed on immune, cardiovascular, and central nervous cells. Adenosine signaling is essential for immune modulation; its extracellular accumulation suppresses pro-inflammatory cytokine production. A2aR expression is mainly observed in T cells, NK cells, NKT cells, monocytes, macrophages, and dendritic cells. Blocking A2aR promotes tumor regression by enhancing T cell cytotoxicity, and combining A2aR inhibitors with PD-1/CTLA-4 inhibitors or adoptive therapy further improves antitumor effects [105,106,107].

BTLA (CD272), typical for Th1 cells, is structurally similar to PD-1 and CTLA-4. BTLA regulates immune responses and maintains tolerance. Its cytoplasmic domain includes three conserved tyrosine motifs essential for phosphorylation and recruitment of phosphatases such as SHP-1 and SHP-2. The BTLA–SHP-1 complex inhibits CD28 and CD3(ε) phosphorylation, thereby suppressing T cell activation. BTLA also supports cell survival, modulates APC function, and influences T cell activation against tumor antigens [108].

TACTILE (CD96), another IgSF member, mediates cell–cell interactions and signaling in immune response regulation. It promotes NK cell adhesion to target cells and regulates cytokine release via binding to CD226 or CD155. Blocking CD96 signaling shows potential in hepatocellular carcinoma, especially when combined with anti-PD-L1 or anti-CTLA-4 therapy. CD96 also affects Th9 cell cytokine production; its inhibition enhances Th9 activity and improves tumor recognition and elimination [109].

SIGLEC-15, a transmembrane protein from the sialic acid-binding immunoglobulin-like lectin (SIGLEC) family, is an immune regulator that suppresses T-cell and macrophage activity through sialic acid-dependent signaling and plays a key role in shaping the immunosuppressive phenotype of tumor-associated macrophages. Its expression is elevated across multiple cancer types, including lung adenocarcinoma, where SIGLEC-15 blockade has emerged as a potential alternative for patients resistant to anti-PD-1 therapy. Mechanistically, interactions between SIGLEC-15-expressing macrophages and sialylated tumor cells promote tumor growth factor beta (TGF-β) production, supporting tumor progression [110].

The main advantages of immune checkpoint inhibitors include oral bioavailability, easy synthesis, and a broad range of targets. Despite the broad therapeutic potential of immune checkpoint inhibitors, their efficacy is frequently limited by resistance mechanisms. One such pathway involves the TIGIT–CD155 axis, which suppresses T-cell and NK cell cytotoxicity and promotes immunosuppressive cytokine production. Co-expression of TIGIT with PD-1 on exhausted lymphocytes and engagement with CD155 on tumor and myeloid cells has been associated with poor prognosis and therapy resistance, highlighting the need for combinatorial checkpoint blockade strategies [111].

One strategy to overcome this limitation is combining CKIs with chemotherapy, radiotherapy, or other immunotherapies. Their efficacy in solid tumors may also be enhanced by applying CCR1/2 antagonists or CXCR1/2 inhibitors, which reduce MDSC infiltration of the TME and inhibit EMT, improving responsiveness to CKIs. Recent studies also highlight bacterial-based therapies, such as *Salmonella typhimurium*, which enhance anti-PD-L1 inhibitor efficacy in colorectal cancer models [112]. Classification of the main groups of immune checkpoint inhibitors according to their primary targets is presented in Table 1.

### 4.3. Oncolytic Virus Therapy

OV therapy relies on the ability of genetically modified viruses to selectively infect and lyse cancer cells through cytopathic effects, simultaneously stimulating antitumor immunity. Its efficacy can be enhanced by combining it with chemotherapy or immunotherapy [92].

OVs trigger antiviral responses via IFN signaling mediated by the JAK/STAT pathway, while phosphatase and tensin homolog on chromosome 10 (PTEN) mutations can disrupt these mechanisms. Their key role is to enhance the recruitment of immune cells to activate anti-cancer adaptive immunity. Viral components such as capsids, nucleic acids, and proteins expose tumor cells to immune recognition, inducing immunogenic cell death pathways (apoptosis, necroptosis, pyroptosis) through endoplasmic reticulum stress. This leads to the release of adenosine triphosphate (ATP), high mobility group box 1 (HMGB1), heat-shock proteins, calreticulin, and pro-inflammatory cytokines (IFNs type I, IL-1β, IL-6, IL-12, TNFα, GM-CSF) as well as chemokines (CCL2, CCL3, CCL5, CXCL10), which attract neutrophils and macrophages to infection sites. Tumor cell lysis further releases tumor-associated antigens presented via MHC class I and II molecules, enhancing T cell activation. Most therapeutic OVs are attenuated viral strains, and resistance remains a major limitation. Therefore, identifying optimal viral vectors and delivery methods is essential. Commonly used OVs include herpes simplex virus (HSV), vaccinia virus (VV), coxsackievirus, adenovirus (AdV), reovirus, measles virus (MV), Newcastle disease virus (NDV), and Sindbis virus [113,114].

HSV has been used in oncolytic cancer therapy since 1991. Currently, seven HSV-based virus types are in clinical use. This virus was engineered by depleting ICP34.5 and ICP47 genes and replacing them with two copies of hGM-CSF. In healthy cells, viral replication is inhibited through protein kinase R (PKR) activation and eukaryotic initiation factor 2 (eIF2) phosphorylation. In cancer cells, disruption of the PKR-eIF2 pathway typically leads to uncontrolled proliferation. ICP47 reduces immune destruction by enhancing MHC1 expression in cancer cells, thereby promoting cancer antigen presentation to T cells [115].

Another HSV-1-based OV, HF-10, belongs to the *Alphaherpesviridae* and naturally lacks expression of several viral genes (UL43, UL49.5, UL55, UL56, and LAT), which weakens the immune response. This virus regulates CD4^+^, CD8^+^, and NK cell populations within the TME, leading to a slight reduction in tumor mass. The therapeutic efficacy of HF-10 has been confirmed in colon carcinoma, peritoneal cancer, and melanoma murine models. HF-10 has been tested both as monotherapy and in combination with immunotherapy (ipilimumab—anti-CTLA-4 monoclonal antibody, or nivolumab—anti-PD-1 monoclonal antibody). Importantly, HF-10 carries natural deletions in UL56 and latency-associated transcript (LAT) and shows high UL53 expression, improving its safety profile [116].

HSV1716, another HSV-based OV, is characterized by infected cell protein (ICP)34.5 deletion. This mutant, derived from the wild-type strain 17, is proposed as a potential treatment for pediatric patients with relapsed or refractory extracranial cancers. It is considered safe for children and young adults with late-stage aggressive tumors, especially high-grade gliomas or refractory non-central nervous system solid cancers. The combination of HSV1716 with immune checkpoint inhibitors offers improved clinical outcomes [117].

G207, another modified strain, was generated through ICP34.5 deletion and ICP6 replacement with *LacZ*. This modification eliminates neurovirulence, while ICP6 attenuation ensures specificity for cancer cells with P16 tumor suppressor defects. The novel M032 is a second-generation HSV1-based OV expressing IL-12, exploiting the observation that dying cancer cells release IL-12, which promotes anti-cancer immune responses. A third-generation HSV1-based virus, G47Δ, resembles G207 but carries an additional ICP47 deletion. Its efficacy has been demonstrated in breast cancer subtypes, while the combination of G47Δ with androgen ablation shows the best results in prostate cancer therapy [118,119].

Adenoviruses represent another group of oncolytic agents. These small, non-enveloped 90–100 nm dsDNA viruses from the *Adenoviridae* family enter cells via interaction between the adenoviral fiber knob and the coxsackievirus–adenovirus receptor. Human adenoviruses are classified into seven species (A–G) with multiple serotypes, among which serotype 5 is most commonly used as a vector. After infection, viral replication induces early gene products (E1A, E1B), leading to host suppressor gene inhibition and cell immortalization [120].

Oncorine (H101), the first recombinant oncolytic adenovirus, was developed for nasopharyngeal carcinoma and is used with chemotherapy. It carries a complete E1B deletion and partial E3 deletion. To date, Oncorine remains the only clinically approved adenovirus-based cancer therapy. ONYX-015 (dl1520), another adenoviral construct lacking the E1B-55 kDa gene, selectively replicates in p53-deficient or p53-mutated cancer cells, while sparing those with wild-type p53. Despite good safety and selectivity, its therapeutic efficacy remains limited [121,122,123].

DNX-2401, an adenovirus-based oncolytic vector, carries a 24 bp deletion in the E1A region and an RGD-4C motif inserted into the fiber knob, enhancing replication and cell infectivity. Similarly, ONCOS-102, engineered to express GM-CSF, is used in combination with pemetrexed/cisplatin or pembrolizumab [124].

Further modifications include the retinoblastoma pathway-selective, hyaluronidase-armed adenovirus VCN-01, applied alone or with gemcitabine and abraxane in refractory retinoblastoma. VCN-01 expresses hyaluronidase, which degrades the extracellular matrix and promotes viral spread. LOAd-703, encoding CD40L and 4-1BBL, is tested in pancreatic cancer either as monotherapy or with gemcitabine for pancreatic, biliary, colorectal, and ovarian tumors. It modulates the TME and activates antitumor immunity. ICOVIR-5 and ICOVIR-7 similarly reduce tumor size [124,125].

The novel adenoviral vector ORCA-010, bearing a T1 mutation (adenine insertion at position 445) in the E3/19K gene, shows high oncolytic potency. This mutation markedly enhances the activity of adenovirus human serotype 5(AdHu5)-based vectors [126,127].

A major limitation of adenoviral therapies is the rapid development of neutralizing antibodies against the vector. This issue may be mitigated by using chimpanzee-derived adenoviruses or less seroprevalent human serotypes (subgroup D) with reduced hepatotropism. Additional strategies include capsid phenotyping, genetic masking via limited heterologous peptide insertion, fiber deknobbing, and PEGylation to evade antibody neutralization [125].

Oncolytic therapy can also employ microvesicles (MVs) from the *Paramyxoviridae* family, typically associated with respiratory infections. Measles-based therapy is considered among the safest, with minimal risk of genomic reversion. Live-attenuated vaccine strains are derived through serial passages in human kidney, amnion, and chicken embryo cells, and originate from the Edmonston strain of measles virus. The MV-EZ (MV-Edm-Zagreb) strain represents a genetically unmodified virus tested in cutaneous T-cell lymphoma (CTCL) patients. Other oncolytic MV variants were engineered to express CEA or human sodium iodide symporter (NIS). The main limitation of measles-based therapy is the presence of anti-measles antibodies that hinder systemic administration. Strategies to overcome this include using cellular vehicles for viral delivery, suppressing innate immune pathways by encoding one or two immune-suppressing genes into MV-Edm, or replacing the H and F glycoproteins with structurally similar but immunologically unreactive analogs from related animal viruses. Combining measles-based therapy with immunosuppressive drugs can also mitigate neutralizing antibody effects [109,110].

Reoviruses are naturally occurring OVs that exploit altered signaling pathways in cancer cells, leading to apoptosis and autophagy. Type 3 reovirus is used in advanced cancers, including refractory brain tumors, in combination with sargramostim, while pelareorep, a naturally occurring live replication-competent type 3 reovirus, is used in combination with paclitaxel to treat patients with recurrent carcinomas [109].

ParvOryx (parvovirus H1) and NDV belong to other naturally occurring OVs. NDV has demonstrated beneficial effects in leukemias, lymphomas, type II and III melanomas, neuroblastomas, fibrosarcomas, colon cancers with liver metastases, post-resection colon cancers, metastatic renal cell carcinomas, and head and neck carcinomas. The specificity of NDV for cancer cells is attributed to defects in the apoptotic pathway in cancer cells. NDV is armed with pro-apoptotic proteins, cytokines, or immunoglobulins, and stimulates anti-cancer response [128].

Cavatak, a naturally occurring picornavirus-based OV (PFVSRIPO), is CD155/Nec15-dependent and contains a modified internal ribosome entry site (IRES) replaced with one derived from human rhinovirus type 2 (HRV2). Pexa-Vec (also known as JX-594), an oncolytic vaccinia virus engineered to express the GM-CSF gene, was tested in patients with recurrent metastatic colorectal cancer in combination with immune checkpoint inhibitors. It was later evaluated in hepatocellular carcinoma, but due to disease complexity, clinical outcomes were disappointing. Another oncolytic vaccinia virus, GL-ONC1, additionally encodes the light-emitting fusion protein Renilla luciferase–Aequorea green fluorescent protein (RUC-GFP) and has been proposed for patients with advanced-stage cancers lacking other treatment options [128].

Vaccinia-derived OV with deletions in the thymidine kinase and growth factor genes, and expressing cytosine deaminase and somatostatin receptor genes, shows potential in treating refractory or metastatic pediatric solid tumors. Additionally, the alphavirus M1, isolated from non-human primates and exhibiting selective replication in cancer cells due to their zinc finger antiviral protein (ZAP) deficiency, has been explored as a therapeutic candidate in cancer patients [129]. Detailed information about characteristics of viruses used in oncolytic virus cancer therapy is presented in Table 2.

### 4.4. Cancer Vaccines

Cancer vaccines are typically used as adjuncts or alternatives to standard anti-cancer therapies. Their main goal is to stimulate the immune system to eliminate tumor cells and induce long-term immune memory [130].

Chemically synthesized vaccines are safe and easy to produce but display low immunogenicity and short-lived immune responses. Nanoengineered vaccines offer superior delivery of antigens and adjuvants to target cells, enhance phagocytosis and antigen processing, and allow surface modification with targeting ligands. Nanoengineering improves serum stability, loading capacity, controlled release, and pharmacokinetic properties. Moreover, nanovaccines enhance antigen cross-presentation by promoting antigen uptake, internalization, and dendritic cell maturation, while protecting encapsulated antigens from proteolysis [90,131].

The application of nanovaccines offers multiple advantages, including controlled release, targeted delivery, improved bioavailability, enhanced penetration into lymph nodes and the TME, high stability, and potent immune response stimulation [132].

A typical nanovaccine consists of antigens, adjuvants, and nanocarriers. Antigens can be tumor-specific antigens (TSAs), expressed only in malignant cells or tumor-associated antigens (TAAs), shared with normal tissues. Adjuvants, which amplify immune responses, may serve as delivery systems (e.g., mineral salts, emulsions, liposomes, virosomes) or immunomodulators (e.g., TLR agonists, stimulator of interferon gene (STING) agonists, co-stimulatory ligands, cytokines). Because adjuvants can cause systemic side effects (fever, nausea, fatigue), their safety requires careful evaluation. Nanocarriers are the key elements of nanovaccines, increasing surface-to-volume ratio, loading efficiency, stability, and biodistribution. They can be biogenic, synthetic, or self-adjuvant [113,114].

Biogenic nanocarriers (e.g., OMVs—outer membrane vesicles, EVs—extracellular vesicles) exhibit high biocompatibility and biodegradability with low toxicity. Semibiogenic carriers, composed of both natural and synthetic components, combine low toxicity with scalable production. Synthetic nanocarriers include liposomes, polymer-based nanoparticles (e.g., PLGA—poly(lactic-co-glycolic acid)), and inorganic materials. Liposomes facilitate degradation and stimulate CTL proliferation, while PLGA-based nanoparticles provide controlled degradation and strong T-cell activation. Inorganic nanoparticles conjugated with TAAs reduce tumor size in murine models. Self-adjuvanted carriers enhance antigen cross-presentation and immune signaling. Some nanomaterials, such as chitosan- or polymethyl methacrylate-based nanoparticles, act simultaneously as adjuvants and nanovaccine components [133,134].

In addition, nanovaccines may incorporate factors such as tumor necrosis factor-α (TNFα), which are capable of directly modulating the TME. TNFα promotes disruption of the endothelial barrier, thereby increasing tumor accessibility and enhancing tumor mass destruction. Furthermore, nanovaccines can be enriched with small-molecule inhibitors or small interfering RNA (siRNA), which not only protect the therapeutic cargo from intracellular degradation but also potentiate antitumor immune responses [33,135].

Some nanovaccines are also designed to deliver inhibitors of angiogenesis, thereby limiting the formation of new blood vessels and restricting tumor growth. Through these mechanisms, nanovaccines can reshape the cellular and molecular composition of the TME and facilitate immune activation. Combining nanovaccines with immune checkpoint inhibition represents a promising strategy to overcome tumor-associated immunosuppression. The inhibition of immune cell control points by the addition of nanovaccine recalls T cells and restores their activity [136].

Cancer vaccines stimulate the immune response through different mechanisms, mainly by using mature DCs isolated from patient blood or generated from monocytes and activated with tumor-specific antigens. After administration, DCs activate immune responses in lymphoid organs and can induce immunological memory, leading to more effective antitumor responses upon subsequent tumor encounter [136].

**Figure 7:**
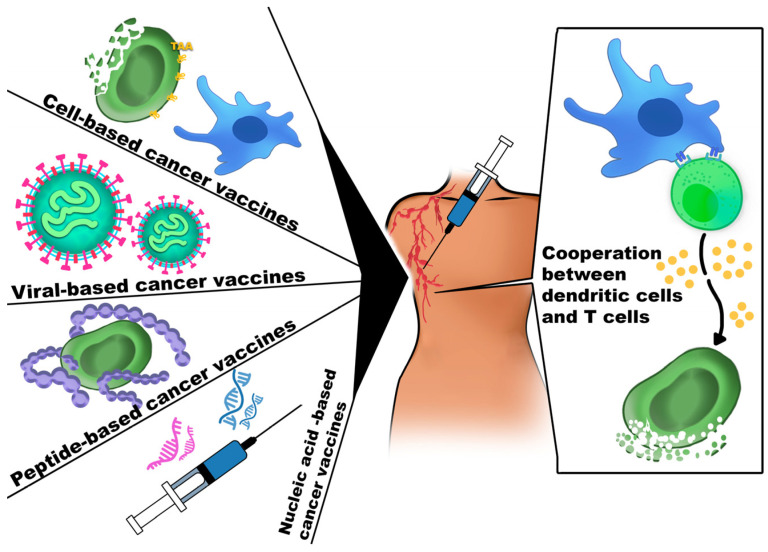
Classification and mechanisms of action of cancer vaccines used in immunotherapy. Cancer cells dying as a result of antigen recognition by T cells and their action.

Cancer vaccines can be prophylactic or therapeutic. Prophylactic vaccines aim to prevent primary or secondary tumor formation and are administered to healthy individuals. Therapeutic vaccines target established tumors by enhancing immune recognition and destruction of cancer cells. They include protein/peptide-based and nucleic acid (DNA/RNA) vaccines. Classical therapeutic examples include Bacillus Calmette–Guérin (BCG) and sipuleucel-T, the latter composed of autologous peripheral blood mononuclear cells (PBMCs) activated ex vivo with a recombinant fusion protein (PA2024) containing prostatic acid phosphatase fused with GM-CSF. BCG is used in asymptomatic metastatic castration-resistant prostate cancer, while sipuleucel-T is applied in most prostate cancer cases [137,138,139]. The mechanisms of cancer vaccines are presented in Figure 7.

Peptide-based vaccines use synthetic short amino acid sequences representing tumor epitopes to stimulate immune responses. They are cost-effective, easily personalized, and safe, as they do not contain live pathogens. Their efficacy relies on antigen presentation by DCs to CTLs and Th cells, leading to coordinated cellular and humoral immune activation [140].

Peptide-based vaccines are limited to patients with compatible MHC types, as only short peptides (<15 amino acids) can activate CTLs. Although their cross-presentation is weaker than that of longer peptides, they are easier to produce and safer. Hepatitis B (HBV) and human papilloma virus (HPV) vaccines are classical peptide-based examples against liver and cervical cancers [141,142].

Nucleic acid vaccines use DNA or RNA sequences encoding tumor antigens to stimulate immune responses. DNA vaccines rely on plasmid-mediated antigen expression and are stable, cost-effective, and capable of enhancing CD8^+^ T cell responses, often requiring adjuvants to improve efficacy. In contrast, mRNA vaccines are translated directly in the cytoplasm, offering faster antigen expression and improved safety profiles [143].

Cell-based vaccines employ whole or fragmented cancer cells, often involving DCs loaded with neoantigens to enhance immunogenicity. DCs are specialized in antigen presentation, and vaccine design depends on the DC subtype used—either monocyte-derived or leukemia cell-derived DCs [144,145].

Cell-based cancer vaccines are generated from inactivated or genetically modified tumor cells and stimulate immune responses against multiple tumor antigens. These vaccines activate dendritic cells, macrophages, and NK cells, leading to coordinated T-cell-mediated tumor elimination [136,146].

In addition, iPSC-based vaccines and in situ cancer vaccines have emerged as novel approaches, promoting broad antitumor immunity through activation of antigen-presenting cells and reprogramming of the TME, including macrophage polarization toward an antitumor phenotype [147,148].

Microbial vector-based vaccines employ viral or bacterial carriers to express and deliver TAAs and stimulate antitumor immunity. Viral vaccines, most commonly based on AdV or VV, selectively replicate in cancer cells, enhancing immune activation and, in some cases, inhibiting angiogenesis; however, their efficacy depends on carefully optimized dosing and scheduling [149].

Bacterial vaccines, using vectors such as *Listeria monocytogenes* or *Salmonella*, primarily stimulate immune responses or deliver immunomodulatory molecules with reduced systemic toxicity, although safety considerations remain important, as some bacteria could be involved in the cancer development (for instance Helicobacter pylori in gastric cancers, or *Neisseria gonorrheae* and *Treponema pallidum* in cervical cancer) [136].

In addition, exosome-based vaccines, derived from tumor cells and enriched in tumor antigens and immunostimulatory molecules, represent an emerging strategy for modulating the tumor microenvironment and enhancing antitumor immune responses [150]. Table 3 summarizes the key characteristics and mechanisms of action of the major types of cancer vaccines.

## 5. Cold Atmospheric Plasma Therapy Directed Against TME-Derived Cells

**Figure 8:**
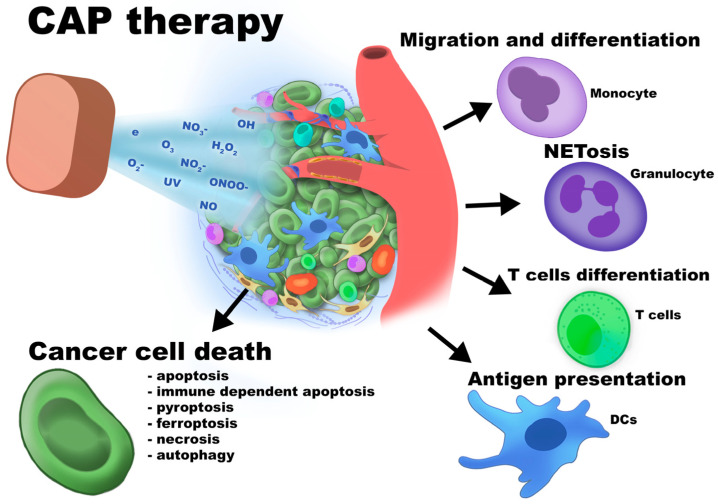
Summary of the mechanisms of cold atmospheric plasma action. For a detailed description, see the main text.

Cold atmospheric plasma (CAP) is a cancer treatment modality based on the application of non-thermal, partially ionized gases. Unlike thermal plasmas, CAP operates at near-ambient temperatures, making it suitable for biomedical use. It consists of a complex mixture of reactive oxygen and nitrogen species (ROS and RNS), ultraviolet radiation, charged particles, and free radicals generated through plasma–air or plasma–liquid interactions [151]. These components collectively mediate the biological effects of CAP on cancer cells. The mechanisms of CAP action are summarized in Figure 8.

The application of CAP in cancer treatment is directly associated with its ability to induce apoptosis, immune-dependent apoptosis, pyroptosis, ferroptosis, necrosis, or autophagy. Briefly, ROS and RNS from CAP can lead to oxidative stress and alter redox balance, which results in cytochrome c releasing and caspases activation. In vitro performed studies revealed enhanced apoptotic gene expression profile in the CAP-treated melanoma and cancer cell lines [152].

CAP can also induce immunogenic cell death (ICD), a regulated form of cell death that stimulates adaptive immune responses through the exposure of dead-cell-associated molecular patterns such as calreticulin, ATP, and HMGB1. This mechanism has been shown to enhance antitumor immunity in several cancer models. In addition, CAP triggers pyroptosis, a highly inflammatory form of cell death associated with mitochondrial damage, caspase activation, and DAMP release [153,154].

Oxidative stress-induced autophagy and necrosis following CAP treatment have been reported mainly in melanoma models, where CAP disrupts membrane integrity, causes ion imbalance, and impairs enzymatic activity. CAP also induces ferroptosis, an iron-dependent form of cell death characterized by lipid peroxidation and membrane damage, as demonstrated in several cancer models [155,156,157].

Importantly, susceptibility to CAP-induced cell death depends on cancer genetic profiles, metabolic states, and TME characteristics. CAP-induced tumor cell death can activate T cell-dependent antitumor immunity by enhancing cancer–immune cell interactions, leading to TME reprogramming, including increased TAM M1 and NK cell activity and suppression of Tregs and TAM M2 populations. Currently, CAP is frequently combined with immunotherapy, chemotherapy, and radiotherapy due to its ability to enhance antigen presentation, sensitize tumors to ROS-inducing agents, and increase oxidative stress-mediated DNA damage. Direct CAP application has been explored in several cancer types; however, indirect delivery approaches have been developed to enable treatment of deep-seated tumors. These include CAP-activated liquids, hydrogels, microneedle patches, and activating devices, each characterized by distinct advantages and limitations related to tissue penetration, stability, and ROS/RNS persistence [151].

Overall, CAP is considered a relatively safe therapeutic approach, as it does not require drug administration and induces cancer cell death through multiple mechanisms that may help overcome resistance to conventional therapies. In addition to stimulating antitumor immunity, CAP remodels the TME by modulating stromal cell function, extracellular matrix organization, and angiogenesis. However, its broader clinical application is limited by the lack of standardized treatment protocols, variability in device configurations, cancer type-specific responses, and incomplete understanding of long-term safety [151].

Given the diversity of cellular and molecular mechanisms that sustain tumor development, no single therapeutic strategy is universally effective across all cancer types. Each cancer type exhibits a distinct TME landscape that shapes both disease progression and treatment responsiveness. Consequently, the molecular targets, therapeutic strategies, and resistance mechanisms described above are different across individual malignancies. The following sections therefore examine how these concepts translate into specific therapeutic approaches in particular solid cancer types.

## 6. The Therapies Directed Against the TME in Various Kinds of Cancers

### 6.1. Head and Neck Cancer Therapies

The heterogeneity of head and neck cancers (HNCs) requires tailoring treatment strategies to each cancer type. Therapy selection depends mainly on the tumor, nodes, metastasis (TNM) stage and the location of the primary lesion. The most effective approaches include surgery, radiation therapy, and chemotherapy, particularly when combined with immunotherapy, which markedly influences the TME. Potentially effective treatment strategy involves reducing doses of the radiation and in several cases omitting or replacing strong chemotherapy by immunotherapy after surgery [158].

One of the examples of it was a combination of chemoradiation and immunotherapy using anti-PDL1 antibodies (Avelumab), although the final results were disappointing. In another clinical trial, monoclonal antibodies were used after chemotherapy; however, the results are still not known. Nevertheless, it is supposed based on the observations that the immunotherapy injected intratumorally could give better outcomes. Mainly, immunotherapy using PD-1/PDL-1 is a strategy each year more often proposed to patients with HNC; besides that, only a limited number of patients positively respond to the PD-1/PDL-1 blockade [159].

The main class of immunotherapy drugs used in HNC treatment is the inhibitors of PD-1/PD-L1. Blocking the interaction between PD-1 expressed on immune cells and PD-L1 expressed on cancer cells allows the CD8^+^ T cells activity to be restored, but the response to the treatment in HNC depends on the HPV status. HPV+ patients are much more sensitive to applied immunotherapy [160].

The second class of the immune checkpoint inhibitors administered to patients with HNC is directed to CTLA-4, TIM-3, LAG-3, or indoleamine-2,3-dioxygenase 1 (IDO-1). IDO-1 participates in tryptophan degradation and Tregs and MDSCs activation and could increase the inflammation in the TME. IDO-1 acts as an immunosuppressant in the TME and is associated with cancer development progression. Low levels of the tryptophan correlate with weak tumor infiltration through NK and T cells and at the same time with poor clinical results [161,162].

It is also possible to apply in those patients co-stimulatory agonists that help in T cells activation and in generation of memory T cells. The most frequently used co-stimulatory agonists are OX40 (CD134), CD40, and glucocortycoid-induced TNFR (tumor necrosis factor (GITR) family-related genes. CD134 is expressed on the CD4^+^ T cells and after binding with OX40L intensifies cancer cell elimination. The expression of OX40 on CD4^+^ T cells is for patients with diminished HNC. The aim of application of an agonist of OX40 is to increase the activity of CD4^+^ and CD8^+^ T cells. Interestingly, the effect of this therapy could be enhanced after combination of OX40 with monoclonal antibodies (anti-PDL-1 or CTLA-4), cytokines, chemical drugs, or radioisotopes [163,164].

In turn, CD40 agonists have currently limited application in HNC treatment, due to their toxicity. Nevertheless, those agonists of CD40 are administered to patients in advanced stages, and their application causes localized immune response activation. This is associated with the fact that CD40 is a critical factor influencing immune cell function and apoptosis. Mostly, CD40 is expressed on the surface of APCs (B cells, DCs, and monocytes/macrophages) and also on the surface of the non-immune cells (endothelial cells, epithelial cells, hematopoietic-derived cells). Anti-CD40 antibodies help in effective anti-cancer immune response owing to the cancer cells survival regulation and successful TAA-antigen presentation [165,166].

GITR that belongs to the tumor necrosis factor receptor family (TNFR) is expressed on the surface of CD4^+^CD25^+^ Tregs, effector cells, or NK cells. The interaction between GITR and its ligands limits Tregs recruitment to the TME of HNC, where it increases the number of T cytotoxic (Tc cells). Therefore, the blockade of GITR helps in immune response regulation. The drugs containing anti-GITR antibodies (for instance AMG228) are well tolerated and have an appropriate level of safety; however, research performed on the animal model has shown better results after using the combination of anti-GITR and anti-PD-1 than after application of only GITR [167].

The popular solution seems to be combining the CKIs with the vascular targeting peptides. This combination allows the increase in the formation of T lymphocytes structure (TLS) and stimulates the T cells activation. The application of the VEGFR2 together with the anti-PDL-1 antibodies shows better results than the application of them separately. This therapy involves high endothelial venules (HEVs) formation, cytolysis, and transformation into the immunosuppressive phenotype. Moreover, possible immunogenic strategies using a combination of T cells therapies with a great harvest of TLSs are the most attractive [159].

One of the main factors influencing the efficacy of immunotherapy in HNC is the inducible T cell co-stimulatory factor (ICOS) expressed on the surface of activated T cells. This ICOS plays an important role in cell signaling, Tregs activation, and CTLs formation. Interestingly, the effect of the agonist of ICOS and pembrolizumab (anti-PD1 monoclonal antibody) was positive—the overall survival (OS) was higher than in patients treated only with anti-PD1/anti-PDL-1 therapy [159,168].

Another opportunity is to administer the anti-CTLA-4 antibodies (durvalumab/tremelimumab), especially in combination with PD-1 and PDL-1 agonists. Such a combination seems to bring favorable effects in patients with HNC who failed platinum-based chemotherapy. The alternative is to apply an anti-PD-1 agonist and IDO, the enzyme implicated in the tryptophan metabolization that limits the activity of CTLs through anergy and suppression induction in them, and at the same enhance Tregs and MDSCs activation. The most well-known IDO antagonist is epacadostat, that, in combination with pembrolizumab, is recommended in patients with advanced stages of HNC [169,170].

Relatively recently discovered B7-H3 agonists (enoblituzumab) bring promising results in patients with HNC, especially when administered together with anti-PD-1 antibody. This is associated with the fact that B7-H3 is involved in negative regulation of Th1 cell function [171].

In HNC, CD24highCD38highCD19+ cells are present at higher density compared with CD19+ B cells in the TME. In tongue squamous cell carcinomas, CD19+ B regulatory cells (Bregs) producing IL-10 promote the differentiation of CD4^+^ T cells into Tregs, which is associated with shorter OS. The prognostic relevance of Bregs depends on their specific phenotype and tissue localization, making them a potential target for immunotherapy [172].

In HPV-positive HNC patients, Bregs expressing CD200+ play a key role in cancer development. Those cells are a potential therapeutic target for samalizumab (the anti-CD200 monoclonal antibody testing in B-cell chronic lymphocytic leukemia (CLL) and myeloma multiplex (MM)). Despite the fact that the final outcomes are not fully satisfied and that the application of antibodies is linked with side effects like skin rashes, joint pain and stiffness, and blood disorders or headaches, the clinical studies using samalizumab are still conducted. The utility of samalizumab is limited additionally by the fact that, in healthy individuals, CD200 is expressed on the normal hematologic and non-hematologic cells—this is the main reason why the Bregs depletion therapy should be always provided with caution. Furthermore, used at the same time as samalizumab, inhibitors of mitogen-activated protein kinase (MEK), Bruton tyrosine kinase (BTK), or signal transducer and activator of transcription (STAT3) sustain Bregs generation and enhance anti-cancer immunity [173].

The antibody ficlatuzumab, targeting hepatocyte growth factor (HGF), inhibits the migration, invasion, and proliferation of head and neck squamous cell carcinoma cells promoted by CAFs. Restoration of miR-124 expression in CAFs and cancer cells in oral squamous cell carcinoma (OSCC) inhibits tumor growth [174,175].

In patients with HNC, a popular therapy is anti-EGFR, which significantly improves the OS. What is worth noting is that not all patients with HNC are predisposed to EGFR therapy (the best clinical outcomes are achieved in HPV-positive HNC patients). EGFR is a tyrosine kinase receptor highly expressed on the surface of the head and neck cancer cells. The high expression of EGFR in patients with HNC correlates with poor prognosis and poor clinical outcomes. The therapies using monoclonal antibodies against EGFR contribute to induction of ADCC and their application reduces cancer progression and is related with a longer survival in those patients. Improved effectiveness of anti-EGFR therapy is achieved in patients with recurrent or metastatic cancer, treated concurrently with pembrolizumab or nivolumab [160,171,176,177].

In this type of cancer, it is also possible to use ACT (tumor-infiltrating T cells or peripheral blood T cells expanded in vitro in the presence of a small part of a tumor). In murine models, ACT combined with IL-2 cytokine gene therapy leads to cancer growth limitation [177].

CAR-MUC1-IL22T used in patients with HNC showed significant cytotoxicity against cancer cells. The efficacy of CAR-T cells was determined against CD70 and neurogenic locus notch homolog protein-1 (NOTCH1) in those patients, and in both situations the clinical outcomes were promising. Nevertheless, the application of ACT in those patients is complicated by HPV positivity, and better results are obtained in HPV-positive patients. Recently, genetically engineered T cells present a novel opportunity for patients with HNC. The most effective are those T cells that are directed against HPV16E6 peptides, probably due to the fact that epitope E6 is highly conserved across all strains of HPV16. In HPV-negative patients with HNC, adoptive therapy included T cells directed against Epstein–Barr virus (EBV) or germline antigens. The latter do not express MHC class I molecules that help in using them for this purpose [178].

### 6.2. Glioma Therapies

In gliomas, immunotherapy is a promising approach which is based on several different methods using checkpoint inhibitors (among them PD-L1 are the most frequently used and, together with IDO-1 and CTLA-4, reduce the number of Tregs and improve OS vaccines), cytokines, CAR-T cells, and monoclonal antibodies. The implementation of immunotherapy is complicated by the fact that the brain is immunologically separated from the blood. However, during glioma growth, the blood–brain barrier may be disrupted, which creates the possibility of using immunotherapy in this group of patients [179,180].

Similarly to patients with HNC, IDO1 is highly expressed in gliomas, and combination therapy with IDO-1 inhibitors and chemoradiation shows beneficial effects in mouse models of glioblastoma. In glioma therapy, cytokines and chemokines such as IL-2, IL-4, and IL-13 may also be used; however, although IL-2 has been evaluated as safe, its combination with vaccination can cause significant side effects that limit its usefulness [181,182].

Prior to molecular classification of glioma-affected patients, the therapeutic strategy in those patients demonstrated unfavorable results. However, the observation of which mutation is present in patients causes better selection of therapy; for instance, administration of bevacizumab gave better clinical results in patients with isocitrate dehydrogenase (IDH1) mutation than in patients without this mutation. Similarly, in patients with diffuse gliomas, the amplification of EGFR and O6-methylguanine-DNA-methyltransferase (MGMT) is associated with better response to immunotherapy, due to the fact that EGFR overexpression is observed in almost 50–60% of glioma patients. However, till now, EGFR tyrosine kinase inhibitors (gefitinib, erlotinib), used in success in lung cancers and in gliomas, have not brought beneficial results. It is uncertain if the failure in gliomas is associated with the brain penetration limitation, escape mechanisms, intratumoral heterogeneity, or resistance development. Despite this, studies evaluating the clinical utility of humanized anti-EGFR variant III chimeric antigen receptor T cells are being conducted [183].

On the other hand, therapy directed against MGMT promotor methylation that serves as a predictor of clinical response to temozolomide is proposed to patients with gliomas. Gliomas expressing BRAFV600E are more sensitive to drugs directed against this mutation. BRAF belongs to the family of the serine/threonine protein kinase, which plays a role in cell differentiation, growth, and proliferation. The BRAF inhibitors are being studied in patients with recurrent gliomas with positive clinical outcomes [184,185].

As far as other immunotherapies applied in gliomas, dendritic cell vaccines are being considered for the treatment of patients with high-grade gliomas. Autologous tumor lysates (ATLs) or glioma-associated antigens (GAAs) are being evaluated as primers for DCs. Final outcomes reveal relative safety and efficacy in gliomas. Therefore, clinical studies using dendritic cells-based vaccines (DCVs) are still being conducted [180].

To date, multiple CAR-T cells have been developed to target gliomas, including those targeting IL-13Ra2, EGFRvIII, HER-2, and CD70. Initial clinical trials showed that using IL-13Ra2 CAR-T cells in glioblastoma is well tolerated and yields beneficial results, encouraging further experiments with second-generation CAR-T cells that incorporate the CD137 molecule alongside IL-13Ra2. Additionally, HER2 CAR-T cells have also shown good tolerance and encouraging outcomes in patients with glioblastoma. Other targets, including EGFR, GD2, ephrin type-A receptor 2 (EphA2), MUC-1, and CD147, are being evaluated, but the final results are still unknown [180].

There is also the possibility to treat gliomas patients through antagonizing the activity of MDSCs that could be performed through the strategies that block their generation and differentiation. In murine models, MDSCs can be depleted using anti-Gr1 monoclonal antibody. However, this therapy has limitations in humans, since MDSCs do not possess unique phenotypical markers; for instance, the most used marker, CD33, is also expressed in other myeloid cells, as well as on activated T and NK cells. Alternatively, sunitinib, a tyrosine kinase receptor inhibitor, can be used to inhibit MDSCs in tumor-bearing mice. Although sunitinib has no direct effect on the anti-cancer response in recurrent gliomas, it could help regulate the immune response [186,187].

IL-4 receptor alpha (IL-4Ra) chain is ubiquitously expressed on the surface of MDSCs and mediates IL-4 and IL-13 signaling. IL-4Ra is crucial for the suppressive activity of MDSCs mediated by arginase (ARG) and TGFβ release. It has been demonstrated that blocking IL-4Ra is a mechanism by which MDSCs can be depleted in patients with glioma. On the other hand, type I interferons may be used in glioma therapies due to their anti-cancer properties, particularly for stimulating MDSC maturation. For instance, IFNα stimulates the conversion of lymphocyte antigen 6G (Ly6Ghigh) cells into pDCs, enhancing anti-cancer immunity [188].

Similarly, arginase inhibitors enhance anti-cancer immunity in glioma patients, both alone and in combination with therapies. Multiple chemical agents, such as hydroxyl-nor-arginine and DL-alpha-difluoromethylornithine, suppress arginase. COX-2 inhibitors can also be considered for glioma patients, especially in combination with retinoids or irinotecan, because MDSCs have receptors for PGE2, and the administration of COX-2 inhibitors reduces MDSC accumulation in tumor-bearing mouse models. The use of COX-2 inhibitors, like celecoxib, is justified in patients with low-grade gliomas due to the high risk of recurrence and progression to high-grade cancer. Multiple cytokines, such as CCL-2 (also known as MCP-1), attract MDSCs toward the tumor mass. CCL-2 is released by glioma cells. Therefore, the application of CCL-2-neutralizing antibodies in murine models of glioma-bearing mice leads to prolonged survival compared to control mice [189].

Targeting the tyrosine kinase BMX, which is highly expressed in pericytes derived from glioma stem cells, may significantly improve the effects of chemotherapy by enhancing the delivery of administered drugs across the blood–brain barrier [190].

### 6.3. Thyroid Cancer Therapies

In thyroid cancers, the primary treatment is surgery, often followed by radiotherapy, thyroid-stimulating hormone (TSH) reduction, or radioactive iodine ablation, depending on tumor stage and risk of recurrence. Beyond standard surgery, radiotherapy, and hormone therapy, modern management increasingly incorporates targeted molecular treatments. This is particularly important in aggressive forms such as anaplastic thyroid cancer (ATC), which show poor response to conventional therapy and frequently carry mutations in BRAF, p53, or rat sarcoma virus (RAS), as well as alterations activating MAPK and PI3K/AKT pathways. These molecular features enable the use of targeted inhibitors, including vemurafenib or dabrafenib for BRAF-mutant cancers. In advanced thyroid cancers, combinations, such as trametinib with pazopanib (a VEGFR tyrosine kinase inhibitor), or MEK1/2 inhibitors like selumetinib, may further improve outcomes, especially in tumors with neuroblastoma RAS viral oncogene homolog (NRAS) mutations [191,192,193,194].

Immunotherapy is an important thyroid cancer treatment that is mainly based on the CTLA-4 and PD-L1 expression observed on the surface of thyroid cancer cells. The expression of both of them in thyroid cancer cells is associated with poor prognosis due to the fact that the expression of CTLA-4 and PDL-1 inhibits T cell activation protecting cancer cells from the activity of immune cells. This is a result of the observation that the high expression of PD-L1 correlates with the high risk of recurrence [195].

Immunotherapy in thyroid cancers is primarily used for advanced stages of cancers resistant to standard therapies. Key immune checkpoint inhibitors in thyroid cancers include pembrolizumab, used in advanced papillary thyroid carcinoma (PTCs) and follicular thyroid carcinoma (FTCs) with PDL-1 overexpression. Moreover, the combined application of pembrolizumab and a tyrosine kinase inhibitor in ATCs with PDL-1 expression contributes to longer patient survival. In these models, tumor size decreased, and survival periods were extended after the administration of anti-PD1/PDL1 antibodies alongside BRAF inhibitors [196].

Lenvatinib, a multi-target tyrosine kinase inhibitor with activity against PDGFR-β, has been demonstrated to impair pericyte viability through suppression of PDGFR-β–MAPK signaling, thereby enhancing thyroid cancer cells death [197].

Motesanib, a multi-tyrosine kinase inhibitor, was investigated in phase II clinical trials in thyroid cancer. Motesanib acts primarily by inhibiting angiogenesis through blockade of VEGF signaling. However, sufficient clinical efficacy was not achieved to justify further development [198].

In thyroid cancer, vaccine therapies targeting New York esophageal squamous cell carcinoma 1 (NY-ESO-1) are particularly used in medullary thyroid cancers (MTCs) that secrete CEA and are being considered as a potential approach, with CEA also viewed as a target antigen in thyroid cancers. Vaccines are potentially useful in ATC due to the observation that these cancers exhibit a high mutation burden, increasing the chances of finding an appropriate antigen. In contrast, OV vaccines contribute to anti-cancer immunity by lysing thyroid cancer cells. They not only suppressed tumor growth in murine models of ATCs but also rechanged TAMs to the TAM M1 phenotype [196,199].

Another approach in the case of ACT involves the administration of cancer-lysate-pulsed DCs in advanced PTC, FTC, and MTC. As for CAR-T cell therapy targeting ICAM-1, also known as CD54, in PTCs and ATCs, tumor growth was effectively suppressed, and patient survival was improved [200].

Anti-ICAM-1 CAR-T cell therapy combined with PD-1-blocking antibodies further limited cancer growth and improved survival in murine xenograft models of thyroid cancers [201].

### 6.4. Esophageal Cancer Therapies

Patients with esophageal cancer (EC) are typically treated with esophagectomy, despite its invasiveness and high risk of morbidity. Post-surgical complications such as appetite loss, dysphagia, aspiration, and reflux have driven the search for less-invasive alternatives. These include endoscopic resection for early-stage disease, neoadjuvant therapy for locally advanced resectable squamous cell carcinomas (SCCs), multidisciplinary management for resectable EC, definitive chemoradiotherapy, salvage esophagectomy, conversion surgery for initially unresectable SCC, and CKI treatment [202].

Neoadjuvant therapy is generally preferred in locally advanced SCC, as surgery alone yields limited results. Novel surgical approaches, including minimally invasive esophagectomy, laparoscopy and thoracoscopy, are increasingly used to reduce postoperative morbidity.

It has been demonstrated that blocking the interactions between CAFs and esophageal cancer cells by targeting IL-6 inhibits tumor growth. PTEN, Akt, MEK, extracellular signal-regulated kinase (Erk), TGFβ1, and CXCL1 are also indicated as potential therapeutic targets due to their association with chemoresistance in EC [203,204,205,206,207].

Immunotherapy in EC is primarily based on activated T cells expressing PD-1 that interact with PD-L1 on tumor cells. PD-L1 overexpression correlates with poor prognosis and adverse clinical outcomes. The introduction of CKIs has improved results in EC treatment. The most commonly used CKIs include pembrolizumab (anti-PD1), recommended as second-line therapy for locally advanced or metastatic EC and as first-line therapy when combined with chemotherapy; nivolumab (anti-PD1) for HER-2-negative advanced EC; and several other anti-PD1 agents such as camrelizumab, sintilimab, toripalimab, and tislelizumab. Anti-PD-L1 antibodies, including atezolizumab and durvalumab, are also used in advanced, recurrent, and metastatic disease. In addition to anti-PD1 therapies, CTLA-4 blockade (ipilimumab) is applied in advanced esophageal adenocarcinoma [208].

Moreover, antitumor vaccines, OV, and adoptive T-cell therapies are considered promising options in EC. Vaccination strategies use neoantigens such as threonine tyrosine kinase (TTK) and NY-ESO-1. Patients receiving peptide-based vaccines show longer disease-free survival [209].

ACT approaches include CAR-T cells and antigen-specific TCR-engineered T cells. Activated lymphocytes have been administered into primary lesions, lymph nodes, or ascites in advanced or recurrent EC. CAR-T therapies targeting HER-2, MUC-1, CD276, or CD70 are under evaluation, with the latter combination enhancing T-cell activation. CAR-NK therapies are less common but CAR-NK cells directed at MUC-1 or HER-2 may also provide benefit. Other candidate antigens include NY-ESO-1, melanoma-associated antigen (MAGE)-A3, and MAGE-A4 [55,210,211].

Clinical trials using autologous tumor-stimulated cytotoxic T cells (AuTLs) have shown benefits, particularly in advanced or recurrent EC. EphA2, highly expressed on EC cells, is another promising target; EphA2-directed CAR-T cells inhibit tumor growth in a dose-dependent manner [212,213,214,215].

### 6.5. Gastric Cancer Therapies

Preoperative treatment of gastric cancers (GCs) is based mainly on chemotherapy, but the development of immunotherapy has brought promising results. Current immunotherapeutic strategies include monoclonal antibodies anti-HER2, anti-PD-1, and anti-PDL-1, although only ~25% of GC patients are HER2-positive. In GC, ubiquitin-specific processing protease 7 (USP7) interacts directly with PD-L1 and stabilizes it; USP7 inhibitors reduce tumor proliferation while promoting PD-1/PD-L1-mediated immune responses [216].

Blocking PD-1/PD-L1 produces partially positive clinical effects. Pembrolizumab is the most commonly used anti-PD1 antibody and is under clinical evaluation for unresectable or metastatic solid tumors, including GC. It is also tested in advanced GC with progression after multiple lines of therapy. Pembrolizumab and nivolumab are evaluated in advanced gastric adenocarcinomas, while the combination of nivolumab and ipilimumab (anti-CTLA-4) shows encouraging outcomes with acceptable toxicity in refractory gastroesophageal adenocarcinoma. Tremelimumab is proposed as second-line therapy for metastatic esophageal and gastric carcinomas. Regulatory markers such as CTLA-4 and FOXp3 show increased expression on CD4^+^CD25high lymphocytes early in treatment, and rapid proliferation of T cells specific for TAAs correlates with improved survival [171,217].

An experimental therapeutic strategy in GC is CAR-T cell therapy, although clinical results are still limited. These approaches aim to restore the function of CD8^+^ T cells, which are often exhausted in GC. CLDN18.2, a gastric-specific isoform of the tight junction protein, is highly expressed on digestive tract cancer cells, making it an attractive therapeutic target. In animal models of CLDN18.2-positive advanced GC, genetically engineered autologous T cells with CLDN18.2-directed CAR (CT401) induced a strong antitumor response. Another promising CAR-T target is CDH17 (LI-cadherin), a cell adhesion protein expressed in the gastrointestinal tract; CAR-T cells against CDH17 eliminated CDH17-positive gastric cancer cells in xenograft models without disrupting intestinal epithelial function [216,218].

Another therapeutic option involves blocking tumor mutation burden biomarker (TMB), as highly mutated tumors generate more neoantigens and show stronger T-cell infiltration [219].

In GC patients with HER2 overexpression or amplification, trastuzumab is used to induce ADCC. Additional targeted treatments include ramucirumab (anti-VEGFR-2), used alone or with PAX; multikinase inhibitors such as lenvatinib and regorafenib; and monoclonal antibodies such as cetuximab (anti-EGFR) and zolbetuximab (anti-claudin 18.2) [219,220].

CAFs produce cytokines that activate the JAK/STAT3 and PI3K/Akt pathways in GC cells, leading to resistance to 5-FU and oxaliplatin. Curcumin inhibits the JAK/STAT3 pathway and reduces the expression of its downstream proteins in CAFs, thereby resensitizing cancer cells to 5-FU. Treatment with calcipotriol, a vitamin D receptor (VDR) ligand, reverses oxaliplatin resistance by counteracting the progressive decline of VDR levels in gastric cancer, as well as in normal and precancerous tissues [221,222].

All-trans retinoic acid (ATRA) suppresses stemness-related genes such as aldehyde dehydrogenase (ALDH), SOX2, and Kruppel-like factor (KLF4) in CSCs, thereby limiting GC progress. CSC inhibition is also observed in liver cancer after treatment with *Celastrus orbiculatus* extract, which deactivates the TGF-β/Smad pathway through inhibition of Smad3/4. MSCs transfected with the suicide gene cytosine deaminase, followed by 5-fluorocysteine administration, can inhibit GC progression [223,224,225].

### 6.6. Pancreatic Cancers Therapies

The poor prognosis in patients with pancreatic cancers (PCa) is the main factor leading to the development of novel anti-cancer therapies. PCa belong to fast progressive diseases; nearly 50–60% of patients display distant metastases, 20–30% with regional involvement, and 10–15% local disease. Systemic chemotherapy is used as palliative treatment in unresectable or metastatic disease and typically includes a combination of 5-FU, folinic acid, irinotecan, and oxaliplatin. Although surgical resection remains the only option offering a meaningful survival benefit, several targeted therapies are also being explored [226].

CAFs play a central role in PCa physiology. Therapeutic strategies directed at CAFs often result in tumor progression rather than elimination, indicating that different CAF subsets may have opposing roles, with some promoting and others restraining tumor growth. Chemoresistance in PCa arises not only from genetic and metabolic alterations but also from interactions with CAFs within the TME. CAFs limit gemcitabine efficacy by forming a physical barrier that reduces drug penetration and by altering gemcitabine metabolism in PCa cells. Consequently, many therapeutic approaches aim either to reduce specific CAF populations or to revert them to inactive states. Other strategies focus on inhibiting key CAF-associated pathways. In mouse models, blockade of JAK2/STAT3 and MEK/ERK1/2 with ruxolitinib and trametinib produced antitumor responses and extended survival [227,228,229,230].

Recent studies have identified a CAF subset expressing leucine-rich repeat-containing protein 15 (LRRC15). Elevated LRRC15 levels correlate with poor response to anti-PD-L1 therapy, and inhibitors targeting LRRC15 are currently under investigation.

FAP, a type II transmembrane serine protease, is another prominent CAF marker. Radioactive tracers targeting FAP show minimal uptake in normal tissues but strong accumulation in tumors. Because CAFs constitute a major stromal component in PCa, Ga68-FAPI demonstrates intense tumor uptake and is emerging as a promising positron emission tomography (PET/CT) diagnostic tool. Ga68-FAPI-PET provides insight into tumor–stroma interactions, potentially advancing understanding of stromal processes in pancreatic cancer [231,232].

Studies have shown that the active metabolite of vitamin (ATRA) restores the quiescent state of pancreatic stem cells (PSCs) through retinoic acid receptor-β (RAR-β)-dependent actin contraction. Returning PSCs to quiescence suppresses their tumor-promoting functions, indicating that retinoic acid-based reprogramming may represent a promising therapeutic strategy in PCa. PSCs can also be activated by oxidative stress via the MAPK/AP-1 pathway mediated by fibromodulin (FMOD), leading to islet fibrosis. In rat models, glutathione inhibits PSC-driven fibrosis by blocking ROS/TGFβ/SMAD signaling. Thus, targeting oxidative stress and PSC activation may be important for limiting fibrosis in pancreatic cancer [233].

One of the most widely explored immunotherapy strategies for PCa is immune checkpoint inhibition. In advanced PCa, the most commonly used CKI is ipilimumab, together with BMS-936559 (anti-PD-L1), which is being tested in patients with advanced disease who have failed previous therapies. Ongoing clinical trials are also evaluating tremelimumab (anti-CTLA-4) administered after 5-FU or gemcitabine-based regimens. In patients with microsatellite stable (MSS) PCa, CKIs used as monotherapy are ineffective and therefore not recommended. This lack of response is likely due to the low immunogenicity of MSS-pancreatic cancer cells. Because CKI monotherapy shows limited efficacy, combination approaches are being explored. For example, durvalumab (anti-PD-L1) combined with tremelimumab is currently under evaluation in metastatic PCa. Other strategies propose combining CKI with radiation therapy to enhance both radiation-induced tumor cell death and T-cell-mediated immune responses [234,235,236,237,238].

Another therapeutic option for patients with PCa is CAR-T cell-based treatment. In PCa, key antigens such as MUC-1, Wilm’s tumor 1 (WT-1), MSLN, and Kirsten rat sarcoma viral oncogene (KRAS) are considered promising targets due to their tumor-specific expression. MUC-1, present in almost all pancreatic cancer cells, enables the use of transfected DCs bearing MUC-1 cDNA, particularly in unresectable or recurrent disease. WT-1, which encodes a zinc finger transcription factor involved in cell growth and differentiation, is expressed in approximately 75% of pancreatic cancers; WT-1 peptide-pulsed DCs combined with chemotherapy have shown favorable effects in advanced PCa. KRAS mutations, present in over 90% of prostate cancer (PC) cases and central to tumor growth and progression, justify KRAS-targeted approaches in these subtypes. Additionally, CEACAM-7 has emerged as a potential CAR-T target due to its expression on pancreatic ductal and epithelial colon cells [239,240,241,242].

Nevertheless, effective CAR-T cell therapy application in PCa remains challenging. Major obstacles include the immunosuppressive TME and the dense fibrotic stroma, which restrict immune cell infiltration. A potential solution is the concurrent use of CAR-T cells targeting pancreatic cancer cells together with CAR-T cells directed against CD19 expressed on B cells. Accordingly, some clinical trials are testing combinations of CAR-T cells with immune checkpoint inhibitors or with OVs engineered to produce pro-inflammatory cytokines to modulate the TME in pancreatic cancers [243].

In patients with PCa, adoptive therapy has also been explored using CTLs generated by isolating T cells from the peripheral blood of healthy donors with the HLA-A24/26 phenotype and stimulating them with MUC-1-expressing human PCa-derived cell lines. Although MUC-1-specific CTLs were shown to effectively eliminate pancreatic cancer cells in preclinical and early clinical studies, the therapeutic benefit was limited. Clinically meaningful outcomes were observed only in patients with resectable tumors, whereas no improvement in OS was reported in patients with unresectable disease, highlighting the restricted efficacy of this approach in advanced cancer settings [244].

Another therapeutic option for patients with PCa is vaccine-based therapy. In a pilot trial, MUC-1-targeted DCs were administered to patients with advanced PCa; however, no clinical benefit was observed. DCVs may be combined with chemotherapy or cytokine-based immunotherapies. Key vaccine targets include RAS, MUC-1, telomerase (GV1001), survivin, G17DT, VEGFR, CEA, WT1, and gastrin [245].

A well-studied DCV is GVAX, composed of allogeneic whole PCa cells expressing GM-CSF, sometimes given with cyclophosphamide (CP) for Treg depletion. In advanced PC, GVAX + CP is also tested in combination with a live-attenuated, mesothelin-expressing *Listeria monocytogenes* strain (CR-207) [246].

Additionally, therapies targeting adipocytes in PCa focus on blocking catabolic pathways and adipokine-dependent signaling. Inhibiting receptors or pathways in cancer cells—such as Notch1 blockade with a leptin receptor antagonist or a γ-secretase inhibitor—may increase sensitivity to gemcitabine or 5-FU [247].

PRI-724, an inhibitor that suppresses CSC self-renewal by targeting β-catenin, is under investigation in PCa and shows efficacy when combined with chemotherapy and immunotherapy. Overexpression of miR-947 has been reported to reduce migration, invasion, metastatic potential, and gemcitabine resistance of pancreatic CSCs via nuclear factor kappa-light-chain enhancer of activated B cells (NFκB1). Additionally, pancreatic cancer growth can be inhibited using gemcitabine-loaded MSCs [248,249,250].

Immune dysfunction in PCa is partly driven by myeloid-derived suppressive cells, and agonistic CD40 therapy promotes macrophage reprogramming toward the TAM M1 phenotype. CCR2 plays a key role in this process, with high expression correlating with poor prognosis; accordingly, a phase I clinical study combining the anti-CCR2 inhibitor PF-04136309 with FOLFIRINOX showed increased OS in nearly half of treated patients. In addition, preclinical studies indicate that combining anti-CSF-1R inhibitors with CKIs may further counteract myeloid-driven immunosuppression in PCa [251,252].

### 6.7. Liver Cancers Therapies

Liver cancer (LC) remains difficult to treat. Surgery, local ablative methods, and liver transplantation are options only for early-stage disease; however, advanced cancers respond poorly to systemic chemotherapy. After resection, adjuvant therapy is recommended to eliminate residual tumor cells and prevent secondary carcinogenesis. Among adjuvant approaches, autologous anti-cancer vaccines and adoptive immunotherapy are of particular interest. Vaccine strategies aim to stimulate antitumor immunity, while adoptive transfer of immune cells provides an external pool of tumor-reactive lymphocytes capable of targeting cancer cells. These immunological approaches are being evaluated alongside standard methods such as transarterial chemoembolization (TACE), interferon and systemic or intra-arterial chemotherapy. The multikinase inhibitor sorafenib also remains an important option due to its activity against Raf-1, VEGFR2/3, FMS-like tyrosine kinase-3 (FLT3), PDGFR, and fibroblast growth factor receptor 1 (FGFR-1) [253,254].

Elimination of CAFs—which contribute to immune evasion, cancer cell survival, and tumor progression—may represent a promising therapeutic strategy. Current CAF-directed approaches in LC focus largely on FAP, with DNA vaccines, adoptive transfer of CAR-T cells, and oncolytic virus-based strategies under investigation for targeting FAP-expressing CAFs [255,256,257].

Immunotherapy for LC also includes CKIs such as ipilimumab, nivolumab, pembrolizumab, and pidilizumab. Their relevance stems from the observation that PD-L1 overexpression correlates with tumor aggressiveness and high postoperative recurrence rates.

Clinical trials with nivolumab showed significant tumor mass reduction and prolonged survival in patients with advanced LC, particularly in those previously treated with sorafenib. In contrast, clinical trials evaluating pembrolizumab yielded less satisfactory results [258].

Another immunotherapeutic strategy involves TILs, as their presence is strongly associated with prognosis in patients with LC. This approach typically uses autologous TILs administered after tumor resection. Although it shows high relative antitumor activity and low toxicity, the number of confirmed clinical responses remains limited [259].

Adoptive transfer of CAR-T cells targeting glypican-4 and alpha-fetoprotein (AFP) is a promising strategy for LC patients. This approach shows particular potential with next-generation CAR-T cells engineered to disrupt PD-1 signaling and co-express ICOSL-41BB, which improves infiltration and antitumor activity in mouse models. Unfortunately, most clinical results involving CAR-T cell therapies are still limited to early-phase trials, and their efficacy has not yet been fully established. In parallel, adoptive transfer of NK cells—derived from stem cells, autologous or allogeneic peripheral blood, or the NK-92 line—prolongs OS and is generally well tolerated, supporting its broader clinical use.

CAR-NK therapy is also under development. CAR-modified NK-92 cells targeting glypican-3 exhibit strong cytotoxicity against hepatocellular cancer cells expressing this antigen. Because TGF-β released by MDSCs suppresses NK cell function by reducing NKG2D and natural cytotoxicity, triggering receptor 3 (NKp30) expression and inhibiting IFNγ secretion, TGF-β represents an additional therapeutic target in LC. Blocking TGF-β restores NK cell activity and enhances their ability to eliminate cancer cells [260,261].

Therapies directly targeting hepatic stellate cells (HSCs) are also under investigation. Studies in animal models show that HSCs can be eliminated using HSC-specific CD8^+^ T cells or CAR-T cells. CAR-T therapy is particularly promising because it selectively removes senescent stellate cells, thereby reducing fibrosis [262,263].

Attention is also given to BiKEs (bifunctional killer engagers), composed of antibody fragments that recognize a tumor antigen and bind CD16a on NK cells. Another strategy aims to enhance cooperation between CD8+ T cells and DCs, showing improved clinical outcomes, better safety, and an increased CD4^+^/CD8^+^ ratio. DC-OK432 vaccines, generated using the streptococcal agent OK432, stimulate strong cytokine and chemokine secretion and enhance T-cell cytotoxicity [258,264].

Although the therapeutic effects of DCVs remain limited, novel preparations such as alpha-fetoprotein peptide-pulsed autologous dendritic cells (AFP-pulsed DCs) are being evaluated. Combining DCVs with CKIs shows encouraging activity, significantly reducing hepatocellular cancer progression. The concurrent use of DCVs and PD-1 inhibitors may also extend OS and reduce tumor burden in liver cancer patients [265,266].

Immunotherapies for hepatocellular carcinoma (HCC) may also target monocytes, particularly through blockade of the CCL15/CCR1 axis. CCL15 is highly expressed on liver cancer cells and recruits CCR1+CD14+ suppressive monocytes to the tumor site, leading to immune suppression and enhanced angiogenesis. Inhibiting this axis reduces tumor growth and lung metastasis. Monocyte reprogramming can also be prevented by targeting key metabolic enzymes involved in shaping the TME [267].

Oncolytic virotherapy is another therapeutic option for LC. Pexa-VEC (JX-594), an oncolytic vaccinia virus expressing GM-CSF, is used to suppress HBV replication and is currently being evaluated in combination with sorafenib. A second approach involves adenoviruses controlled by cancer-specific promoters to achieve selective tumor targeting. The human telomerase reverse transcriptase (hTERT) promoter, highly active in cancer cells but largely inactive in normal tissues, enables tumor-specific viral gene expression. OBP-301 is a replication-competent adenovirus in which the hTERT promoter drives selective viral replication, leading to lytic death of cancer cells while sparing healthy tissues. Preclinical in vitro and in vivo studies have demonstrated effective tumor cell lysis, antitumor activity after intratumoral administration, and minimal systemic toxicity [268,269].

### 6.8. Colorectal Cancer Therapies

Classically, non-metastasized colorectal cancer (CRC) is treated surgically, with procedure type and timing determined by tumor stage and patient condition. In rectal cancer, early-stage tumors benefit from surgery, and recurrence risk can be reduced with short-course radiotherapy or neoadjuvant therapy in unfavorable cases [270].

In metastatic CRC, standard chemotherapy typically uses combinations of 5-FU, leucovorin, oxaliplatin, or irinotecan, with capecitabine as an alternative to 5-FU [271].

Another therapeutic approach is targeted therapy, used primarily in metastatic CRC. Four main groups of agents are applied: monoclonal antibodies against EGFR (cetuximab, panitumumab), anti-VEGF-A antibodies (bevacizumab), fusion proteins targeting multiple proangiogenic growth factors (aflibercept), and small-molecule multikinase inhibitors (regorafenib). Nearly 80% of CRCs express or overexpress EGFR, and its expression correlates with shorter survival and increased metastasis risk. Anti-EGFR monoclonal antibodies improve progression-free survival in metastatic disease. Conversely, RAS mutations, present in almost half of CRCs, render Ras GTPase constitutively active and drive tumorigenic signaling pathways [272,273].

Angiogenesis in CRC is driven by VEGF-A secretion and VEGFR-1/VEGFR-2 activation. Anti-angiogenic therapies such as bevacizumab and aflibercept (a VEGFR-1/VEGFR-2 fusion protein linked to IgG1 Fc) are widely used in metastatic CRC [274].

**Figure 9:**
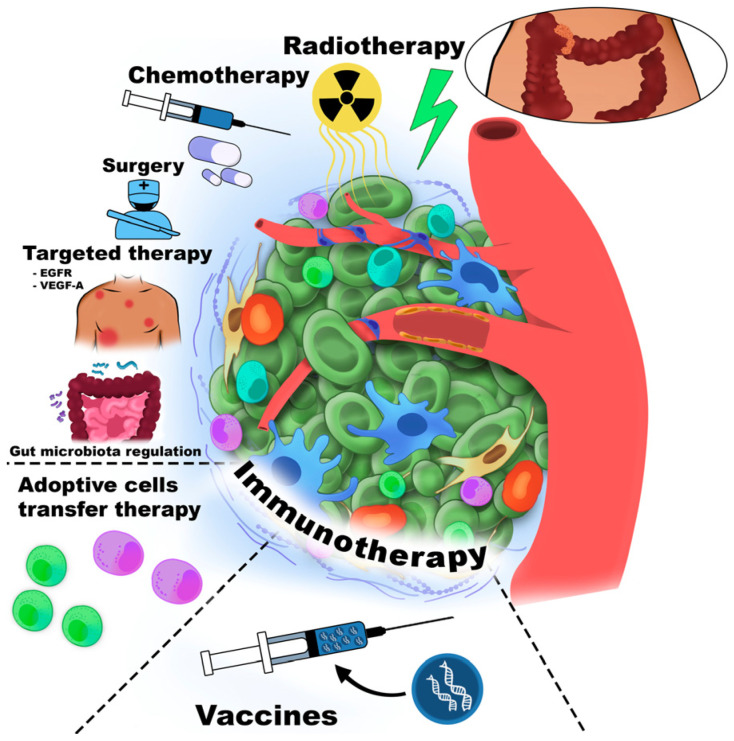
Common treatment strategies used in patients with colorectal cancers.

Immunotherapy in CRC aims to enhance CD8^+^ T-cell infiltration and limit T-cell exhaustion through multiple mechanisms, including modulation of protein expression (e.g., apolipoprotein—APOL3, microtubule-associated protein—MAP7D2, FMOD, tribbles homolog 3—TRIB3, guanylate-binding protein 2 (GBP2)—GTP enzyme family), immune checkpoint inhibition, cancer vaccines, modulation of intestinal flora, RNA-based regulation, and MSC-based therapies. APOL3 regulates ferroptosis-associated CD8^+^ T-cell infiltration and, together with lactate dehydrogenase A (LDHA), promotes tumor cell degradation. MAP7D2, highly expressed in MSS CRC cells, influences CD8^+^ T-cell responses. FMOD, a member of the small leucine-rich repeat proteoglycan (SLRP) family, contributes to signaling and matrix organization, while TRIB3 is involved in colon cancer development [275]. Figure 9 presents the most popular treatment strategies used in patients with CRC.

The most widely used form of immunotherapy in CRC is CKIs. However, primary immunotherapy against CTLA-4 (tremelimumab) has shown limited efficacy in treatment-refractory colorectal cancers, and similar limitations have been reported for anti-PD-1/anti-PD-L1 therapies. Only the introduction of mutation testing into CRC diagnosis has led to improved clinical outcomes [276].

Approximately 15% of CRCs display microsatellite instability (MSI). Despite variable responses to chemotherapy, patients with MSI generally have a better prognosis. Dual checkpoint blockade with nivolumab and ipilimumab is frequently used in MSI tumors and provides beneficial results [276,277].

Pembrolizumab and nivolumab have been approved for mismatch repair (MMR)-deficient (dMMR) and microsatellite instability-high (MSI-H) colorectal cancers. Because improved outcomes are often linked to mutational load, patients with dMMR-MSI-H tumors and high numbers of mutations may particularly benefit from vaccination [278,279].

In an open-label, randomized phase III clinical trial evaluating the efficacy of nivolumab plus ipilimumab compared with nivolumab monotherapy and standard chemotherapy combined with bevacizumab in patients with MSI-H CRC, the combination therapy reduced the risk of disease progression or death by nearly 80% [280].

Several antigen-based vaccines are being developed for CRCs. Viral-vector vaccines using chimpanzee adenoviruses or Venezuelan equine encephalitis virus induce strong CD8^+^ T-cell responses. PolyPEPI1018 contains six synthetic peptides and twelve epitopes derived from seven conserved cancer testis antigens (CTAs). CTAs play important roles in metastasis, drug resistance, and the maintenance of cancer stem cells. These antigens are aberrantly overexpressed in many cancers while being silenced in most normal tissues, which makes them attractive and highly specific targets for cancer immunotherapy. Other candidates include guanylyl cyclase C (GCC) vaccines, which selectively stimulate cytotoxic CD8^+^ T cells, and OncoVAX, which uses autologous cancer cells supplemented with TAAs to prevent recurrence after surgery. Vaccine efficacy is typically enhanced when combined with CKIs [281,282].

Finally, adoptive cell therapy, including CAR-T cells and TILs, may be used in CRC. Early studies show encouraging results. CAR-T cells are primarily directed against CEA, which is minimally expressed in normal tissues but abundant in CC cells [283].

Another promising strategy to enhance anti-cancer immunity is the combination of anti-PD-L1 therapy with NKGD2 receptor CAR-T cells. Preclinical studies of NKG2D- and DAP12-targeted CAR-T cells demonstrated enhanced NK cell activity and antitumor efficacy in murine CRC models, with preliminary clinical observations supporting their potential in humans. Notably, CYAD-101, a non-gene-edited NKG2D-based CAR-T therapy, showed encouraging clinical activity in patients with metastatic colorectal cancer (mCRC) refractory to oxaliplatin [284].

Encouraging results have also been observed with TILs—polyclonal CD8^+^ T cells targeting mutant KRAS G12D and readministered to patients. A major goal of immunotherapy in CRC is to improve CD8^+^ T-cell infiltration and limit T-cell exhaustion. This can be achieved through modulation of protein expression (e.g., apolipoprotein, microtubule-associated protein, fibromodulin, tribbles homolog 3, guanosine triphosphate (GTP) enzyme family), inhibition of immune checkpoints, RNA modifications, and the use of MSCs [281].

Gut microbiota also influence immunotherapy efficacy. A newly developed probiotic formulation containing *Lactobacillus plantarum*, *Bifidobacterium*, and polysaccharides has been shown to reduce Treg levels and increase CD19, CD4, and CD8 lymphocytes. *Bifidobacterium* and *Akkermansia municiphila* may enhance the activity of CKIs through inosine–adenosine A2 receptor (A2AR) signaling. Inosine, an endogenous nucleoside formed by metabolic deamination of adenosine, exerts anti-inflammatory and immunomodulatory effects. T-cell-specific inosine–A2AR signaling appears to be a promising therapeutic mechanism that acts synergistically with other immunotherapies, as gut microbiota and TILs can jointly remodel the TME and improve treatment outcomes [208,285].

It has been shown that anti-CD24 monoclonal antibodies targeting the CSC surface marker CD24 inhibit CRC growth. In addition, miR-215 regulates the self-renewal and multipotency of colorectal CSCs as an effector of CDX1 Caudal Type Homeobox 1 (CDX1) and a repressor of B cell-specific Moloney murine leukemia virus integration site 1 (BMI1) proto-oncogene, polycomb ring finger [286,287].

The activity of sibrotuzumab, a monoclonal antibody targeting FAP, was evaluated in colorectal cancer. However, in a phase II clinical trial, it failed to meet expectations due to a lack of significant efficacy [288].

### 6.9. Lung Cancer Therapies

**Figure 10:**
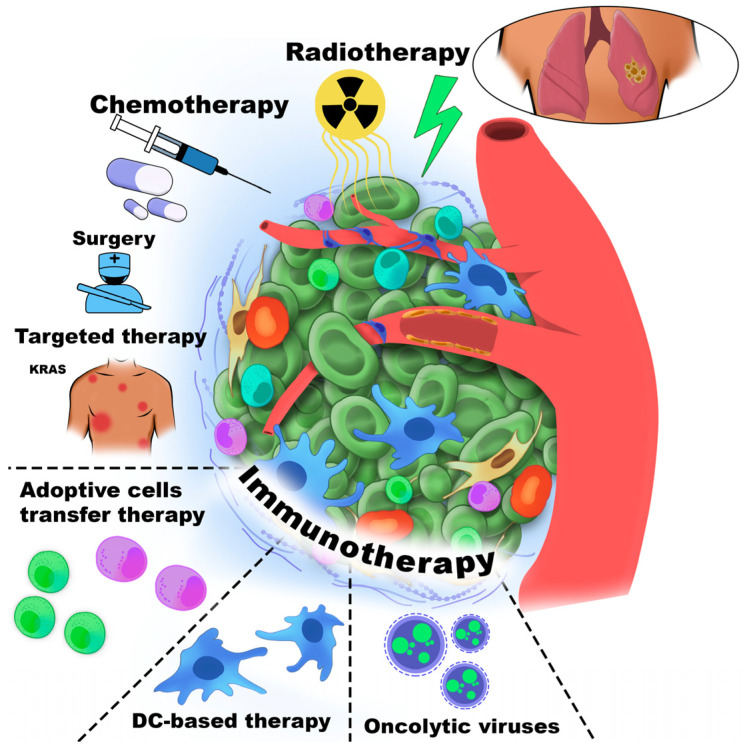
Common treatment strategies used in patients with lung cancers.

The primary treatment methods for lung cancers, including both non-small cell lung cancer (NSCLC) and small-cell lung cancer (SCLC), are surgery, cytotoxic chemotherapy, and radiotherapy. However, cure rates remain low because resistance to therapy is frequent. As a result, new therapeutic targets are being explored, particularly those associated with cells in the cancer microenvironment [289,290,291]. An overview of the most common treatment strategies used in lung cancer is presented in Figure 10.

The IL-6/JAK2/STAT3 pathway contributes to cancer cell survival, growth, metastasis, and therapy resistance. Accordingly, inhibitors of this pathway are being investigated to disrupt interactions between CAFs and cancer cells. Inhibiting myofibroblastic cancer-associated fibroblasts (myCAFs) by targeting plasminogen activator inhibitor-1 (PAI-1) reduces cisplatin resistance in lung cancer [134,292,293].

Tyrosine kinase inhibitors (TKIs) such as nintedanib, motesanib, and sorafenib block VEGF receptors 1–3, FGF receptors 1–3, and PDGF receptors α and β. Nintedanib is approved in combination with docetaxel for the treatment of advanced NSCLC following first-line platinum-based chemotherapy [294].

Only a minority of NSCLC patients respond to CKI therapy, although these agents are widely used in advanced lung cancer. For PD-L1-positive patients after prior chemotherapy, pembrolizumab is applied as first-line therapy, while atezolizumab is approved for advanced NSCLC, progressing despite standard treatment [269,295].

Pembrolizumab has also been tested in advanced SCLC, where it doubled overall response rate (ORR) and was well tolerated, showing benefits even in PD-L1-negative disease. In advanced NSCLC with PD-L1 expression, pembrolizumab improves progression-free survival (PFS) and OS compared with standard chemotherapy and is generally safe, with fatigue, pruritus, and appetite loss as the most common adverse effects [296,297].

Pembrolizumab may also be combined with docetaxel after platinum therapy, improving OS and PFS. Additionally, pembrolizumab with etoposide is recommended as first-line therapy for untreated extensive-stage small lung cancer (ES-SCLC). Untreated metastatic NSCLC can be managed with pembrolizumab combined with chemotherapy (carboplatin + paclitaxel/nab-paclitaxel) [298,299].

Patients with NSCLC respond better to adoptive cells transfer therapy, particularly TIL therapy, than patients with other lung cancer types. TIL-based therapy after lung resection has shown clinical benefit in small tumors, and combining TILs with nivolumab induces tumor regression, although without a clear OS improvement. ACT strategies in lung cancer include CAR-T cells, TIL therapy, TCR-based therapy, and NK cell therapies.

CAR-T cells targeting CEA and MUC-1 are the most frequently used, and CD276-directed third-generation vaccines also show promise [300,301,302].

NK cell-based therapies aim to enhance NK cell proliferation, prevent suppression, and improve tumor recognition. Approaches include monoclonal antibodies with cytokines, PBMCs activation with IL-12/IL-2, and IL-15-based strategies. Mixed NK cell–DC adoptive therapy has shown benefit in murine models, and combining NK cells with T cells may improve OS in NSCLC [303,304].

Vaccines for NSCLC, valued for low toxicity, target proteins such as EGF, MAGE-A3, NY-ESO-1, and MUC-1. EGF-dependent vaccines improve survival; MAGE-A3 and NY-ESO-1, expressed in 30–50% and ~30% of NSCLC, respectively, are associated with better prognosis; and MUC-1 vaccines are under evaluation as maintenance therapy. However, a phase III study evaluating a recombinant protein vaccine composed of MAGE-3 and fusion protein D derived from *Haemophilus influenzae* failed to demonstrate an improvement in disease-free survival (DFS) compared with the control group [305,306,307,308]. In addition, two other allogeneic vaccines, Tergenpumatucel-L and Viagenpumatucel-L, are currently being investigated in combination with CKIs, as well as tecemotide, a liposome-based vaccine derived from MUC-1. In a cohort of 1513 patients with unresectable NSCLC treated with tecemotide combined with either concurrent or sequential chemoradiotherapy, an improvement in overall survival was observed only in patients receiving concurrent chemoradiotherapy. A confirmatory trial of tecemotide in patients with advanced NSCLC following concurrent chemoradiotherapy is currently ongoing [309].

OVs are also under investigation for their ability to modulate the TME in NSCLC. HSV-1 has shown beneficial effects in human NSCLC-derived cell lines, while Coxsackievirus B3, adenovirus rAD-p53, and Seneca Valley virus isolate 001 are likewise being evaluated [295].

Inhibition of the protumorigenic activity of CSCs in lung cancer can be achieved with hydroquinone 5-O-cinnamoyl ester of renieramycin M, which suppresses PI3K/AKT and c-Myc activity [310].

First-line therapy for SCLC is based on etoposide–platinum regimens, often combined with atezolizumab or durvalumab. To increase the proportion of patients benefiting from immunotherapy, CKIs are increasingly paired with non-chemotherapy agents. One strategy involves restoring the balance between immune activation and suppression using nivolumab and ipilimumab. Another relies on enhancing the immune response through PD-1/PDL-1 blockade combined with chemotherapy. Utomilumab, a fully human IgG2 agonist antibody targeting CD137, further boosts T cell and NK cell cytotoxicity [311].

Additional co-stimulatory agents strengthen TCR signaling. Examples include INCAGN01876, which binds GITR, and INCAGN01949, which targets OX40; both enhance T cell priming, effector differentiation, and memory-cell renewal [312].

Antigen-specific vaccines are another therapeutic option, particularly after chemotherapy. SCLC cells express GD3, enabling the use of BEC2 (anti-idiotypic monoclonal antibodies mimicking GD3), which increased OS in nearly 40% of treated patients. Other potential vaccine targets include GM2, globo H, fucosyl GM1, and polysialic acids, which are selectively expressed on SCLC cells. Finally, p53 represents another relevant antigen. Patients receiving a p53 vaccine combined with chemotherapy demonstrated high overall response rates [313,314,315,316].

### 6.10. Breast Cancer Therapies

In breast cancer (BC), treatment relies on multimodal strategies combining neoadjuvant therapy, surgery, radiotherapy, and endocrine therapy. Neoadjuvant therapy is recommended for locally advanced or inoperable tumors and increasingly used in early stages to improve surgical outcomes. Adjuvant treatments include irradiation, cytotoxic drugs, targeted agents, and hormones. Hormone therapy is essential for all receptor-positive BC, with tamoxifen used in both pre- and postmenopausal patients. Neoadjuvant hormone therapy is proposed for hormone receptor-positive (HR+) tumors and may be given with radiotherapy, though not with chemotherapy [317,318].

Conventional BC chemotherapy uses alkylating agents (e.g., cyclophosphamide) and antimetabolites (methotrexate, 5-FU), reducing recurrence risk. Adjuvant therapy is given after surgery, while neoadjuvant therapy precedes it. In TNBC, pembrolizumab combined with niraparib is recommended, particularly in breast cancer gene (BRCA)-mutant tumors. Pembrolizumab with chemotherapy is also used as first-line TNBC treatment, as well as in neoadjuvant–adjuvant regimens [319,320,321,322].

**Figure 11:**
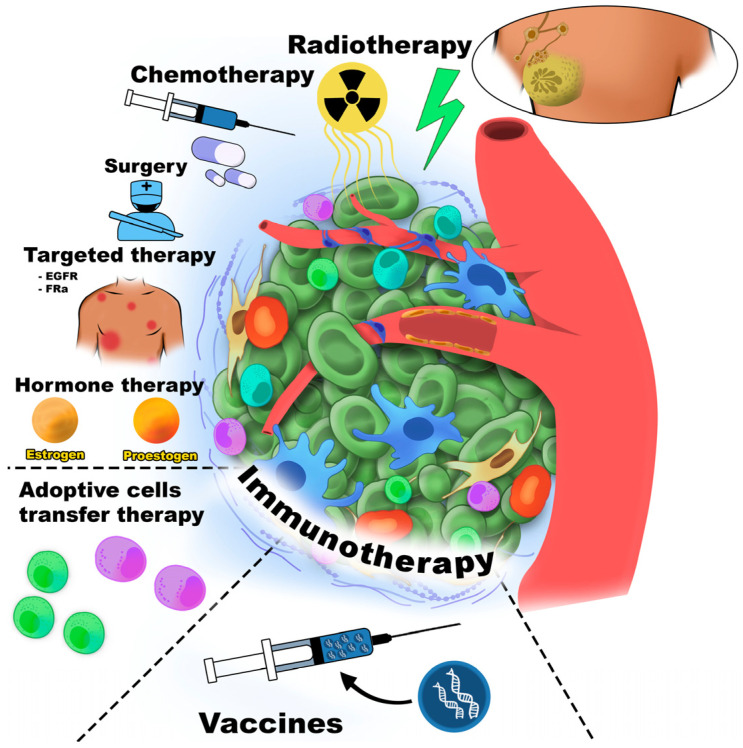
The principal treatment strategies currently used in patients with breast cancers.

Adjuvant hormonal therapy aims to prolong OS by limiting metastasis. Premenopausal estrogen-positive patients receive tamoxifen, sometimes with a luteinizing hormone-releasing hormone (LH-RH) agonist in high-risk disease. Postmenopausal patients at high risk are treated for up to 10 years, while lymph node-positive cases typically receive anthracyclines or docetaxel plus cyclophosphamide. Anti-HER-2 therapy (trastuzumab) is used in HER2+ cancers and tumors > 1 cm, while pertuzumab further improves DFS in high-risk cases [323,324]. The main therapeutic approaches applied in patients with breast cancers are illustrated in Figure 11.

Drugs targeting CAFs represent a promising therapeutic strategy in BC. TGF-β is a key mediator of CAF–cancer interactions, and both TGF-β receptor inhibitors and TGF-β-neutralizing antibodies have shown the ability to suppress tumor progression. Fresolimumab, a monoclonal antibody, neutralizes all three TGF-β isoforms. Galunisertib, a TGF-βRI inhibitor, enhances T cell activity and inhibits tumor growth when combined with PD-L1 blockade in mouse models [325,326].

Hyaluronic acid (HA), produced mainly by CAFs, contributes to BC malignancy. Pegvorhyaluronidase alfa (PEGPH20) remodels the TME by degrading HA, improving drug delivery and increasing anti-PD-L1 uptake [327,328].

FAP-based vaccines targeting CAFs are under investigation and have been shown to increase IFN-γ production, expand CD8^+^ T cells, reduce collagen I levels, and improve drug penetration [329,330].

Histone deacetylase 6 (HDAC6), frequently upregulated in CAFs and associated with poor prognosis in BC patients, is another potential target. HDAC6 inhibition reduces tumor growth, prevents monocyte and Treg accumulation, and activates CD4^+^ and CD8^+^ T cells [331,332].

Losartan inhibits angiotensin IL-1 receptor signaling in CAFs, decreasing TGF-β, cellular communication network factor 2 and endotelin-1 expression, reducing collagen and hyaluronan deposition, and enhancing drug and oxygen delivery. Its combination with camrelizumab and liposomal DOX is currently being investigated in BC patients [333,334].

The Hedgehog pathway, which activates CAFs and promotes ECM remodeling and docetaxel resistance, is another therapeutic target; inhibition with sonidegib plus docetaxel has shown promising activity [335].

Finally, FGF and CXCL signaling contributes to BC cells proliferation, migration, and therapy resistance. FGFR inhibitors such as erdafitinib, AZD4547, and futibatinib suppress BC cell growth and overcome resistance mechanisms. ATRA can shift CAFs into a dormant state, reducing their pro-tumor activity [336,337,338,339].

As in other cancers, therapies in BC also target immune checkpoints. Blocking the PD-1/PD-L1 axis or CTLA-4 yields positive effects in BC patients. Sensitivity to immunotherapy is influenced by hyaluronan accumulation, and its degradation by TME-remodeling enzymes increases responsiveness to anti-PD-L1 therapy—an approach proposed for metastatic TNBC, although its broader usefulness requires further evaluation. To date, pembrolizumab is the only immune checkpoint inhibitor officially approved for BC, with indications restricted to TNBC. Nevertheless, pembrolizumab monotherapy in patients with previously treated metastatic TNBC has not shown significant increase in OS as compared to chemotherapy. Interestingly, some studies suggested that chemotherapy can increase the anti-cancer effect of CKIs. Combination strategies pairing pembrolizumab with standard treatments are being explored for additional BC subtypes [340].

Immunotherapy benefits many TNBC patients, yet resistance to PD-1/PD-L1 inhibitors still occurs. Resistance mechanisms include extrinsic factors related to the TME (altered CD8^+^ T cells, Tregs, MDSCs, TAMs) and intrinsic factors such as dysregulated oncogenic signaling, cytokine/chemokine imbalance, and impaired immune checkpoint regulation. PARP inhibitors, which target poly(ADP-ribose) involved in DNA repair, may help overcome such resistance [340,341,342].

CAR-T cell therapy in TNBC is mainly directed against MUC-1, NKG2D, AXL receptor tyrosine kinase (AXL), c-Met, and MSLN. Promising results have been reported in early-phase clinical trials using c-Met–CAR-T cells. However, clinical studies of CAR-T cell therapy in TNBC remain limited, largely because many target antigens are also expressed in healthy tissues, increasing the risk of toxicity [343].

More commonly used in BC patients is TIL therapy, which correlates with improved clinical outcomes. TNBC and HER2+ subtypes respond particularly well. ACT in BC often utilizes CD57+ NK cells, whose cytotoxic activity (via CD16) and intratumoral abundance serve as biomarkers of response to HER2-directed therapies and overall prognosis [341,344].

BCs responding to trastuzumab typically show increased NK cell infiltration and enhanced ADCC. CAR-NK cell therapy is emerging as a promising option, especially in HER2+ disease. Treg depletion using anti-CD25 monoclonal antibodies or pentoxifylline can further improve IL-2-mediated TIL expansion. In contrast, CAR-T cell therapy is most effective when directed against MUC-1, expressed in ~95% of BC, and shows greatest benefit in metastatic disease [345,346,347,348].

Vaccines in BC aim to induce or amplify T-cell responses against tumor antigens. Platforms include peptide vaccines, DNA vaccines, and DC vaccines. Studies comparing the safety profiles of two peptide-based anti-HER2 cancer vaccines (GP2 and AE37) indicate that both are safe, but their efficacy depends on breast cancer subtype. AE37 showed clinical benefit mainly in TNBC patients with low HER2 expression, whereas the GP2 vaccine was more effective in patients with HER2 overexpression. These findings highlight HER2 expression as a key determinant of therapeutic response in this type of immunotherapy [349].

STn-targeted vaccines, directed against the sialyl-Tn carbohydrate on MUC-1, have shown tumor regression in animal models. Additional strategies include poly-antigen vaccines secreting GM-CSF to enhance NK cell cytotoxicity and viral-vector vaccines delivering CEA, MUC-1, and T-cell-stimulating molecules, though clinical benefit remains modest. Alternative vaccine concepts involve mutated cancer antigens, bispecific small molecules (e.g., CD3 and HER2 or CD3 and p-cadherin), and dual affinity retargeting technology (DART) molecules targeting dual checkpoints such as PD-1/CTLA-4 or PD-1/LAG-3 [341,350,351].

B cells within tumor-infiltrating B cells (TIBs) produce IgG directed against cancer antigens and may serve as therapeutic targets in BC. A high number of TIBs correlates with better outcomes, whereas increased Bregs with immunosuppressive activity is unfavorable; therefore, strategies aimed at Bregs depletion are being explored [352].

Therapeutic approaches in BC may also target adipogenesis and adipose-derived signals that support tumor growth. Adipogenesis regulatory cells (Aregs), characterized by high CD142 expression, inhibit adipocyte differentiation and represent a potential therapeutic target. Adipogenesis may also be suppressed by sulforaphane, which enhances MSC self-renewal. In addition, leptin peptide receptor antagonist 2 (PEG-LPrA2) inhibits estrogen receptor (ER; ER+/ER− BC) growth by suppressing VEGF, suggesting its utility in therapy. Various anti-inflammatory agents, including ibuprofen, mefenamic acid, celecoxib, and diclofenac, are also considered for targeting adipocyte-related pathways [353,354,355].

Compounds such as curcumin and epigallocatechin-3-gallate (EGCG) inhibit CSC pro-tumorigenic activity by blocking JAK/STAT and NF-κB signaling. Other agents, including PdCl(terpy)_2_H_2_O–niclosamide complexes, suppress the Wnt pathway and induce CSC apoptosis in BC [356,357].

Polymeric nanoparticles targeting CD133 demonstrate tumoricidal effects in BC. Additionally, MSCs loaded with PTX inhibit tumor growth by reducing proliferation and inducing apoptosis, while MSCs loaded with DOX also show cytotoxic activity against BC cells [358,359,360].

### 6.11. Ovarian Cancer Therapies

**Figure 12:**
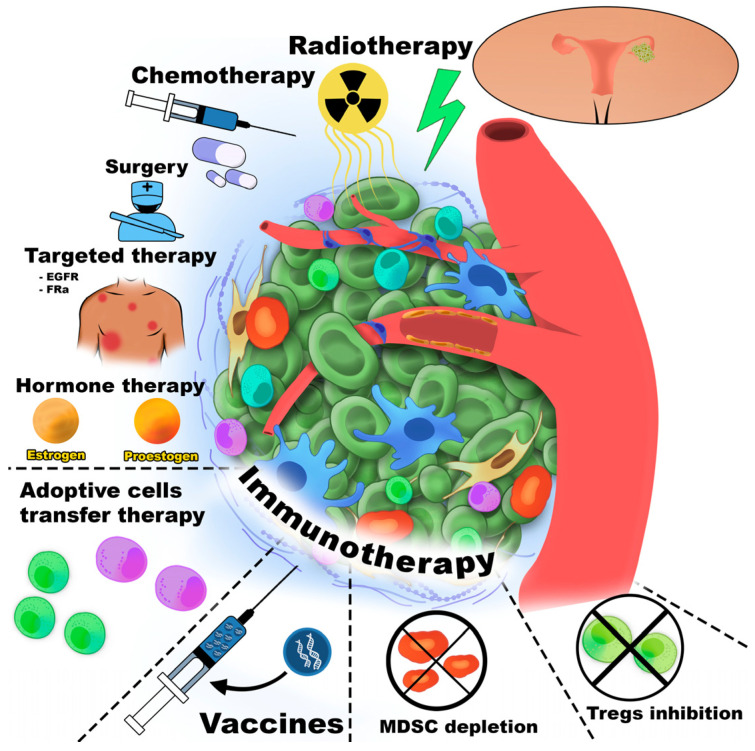
Common treatment strategies used in patients with ovarian cancers.

Standard therapy for ovarian cancer (OC) includes maximal cytoreductive surgery followed by platinum-based chemotherapy. Early-stage patients undergo surgery with intravenous platinum/taxane, whereas advanced stages are treated with neoadjuvant chemotherapy followed by interval surgery if response is achieved. OC management also includes bevacizumab and other anti-angiogenic agents (cediranib, pazopanib, aflibercept), commonly combined with PTX, pegylated liposomal doxorubicin (PLD), or topotecan, particularly in recurrent epithelial OC. Hyperthermic intraperitoneal chemotherapy (HIPEC) with cisplatin or taxanes is another option in selected patients. PARP inhibitors (olaparib, niraparib, rucaparib, veliparib, talazoparib) are widely used in recurrent BRCA-mutated OC and are generally well tolerated. EGFR tyrosine kinase inhibitors (erlotinib, cetuximab, lapatinib) may be considered in patients with high EGFR expression, which correlates with shorter DFS and OS. Folate receptor alpha (FRa), a glycosylphosphatidylinositol-anchored protein, is commonly overexpressed in epithelial OC. FRa expression can be targeted with specific antibodies or antibody-like binders such as farletuzumab or mirvetuximab soravtansine. FRa also serves as a promising target for cancer vaccines and T cell-based immunotherapies [361,362,363,364,365]. Figure 12 summarizes the principal treatment strategies currently used in patients with ovarian cancers.

In OC, normal fibroblasts are reprogrammed into CAFs through decreased expression of miR-31 and miR-214 and increased expression of miR-155. CCL5, a direct target of miR-214, can inhibit tumor growth and reduce metastatic potential. CAFs in OC are resistant to PARP inhibitors (PARPi); PARPi activate stromal fibroblasts by increasing CCL5 secretion via NF-κB signaling. Thus, CCL5 neutralization may shift CAFs from an activated to a quiescent state. Blocking CAF activation can also be achieved by targeting TGF-β, for example, through CXCR4 downregulation and TβR–Smad inhibition, which disrupts the TGF-β/SDF-1 feedback loop. Additional CAF-related targets, such as MYC and VCAN, are under investigation. Bevacizumab inhibits CAF infiltration and progression of OC. Bevacizumab is the only approved anti-angiogenic agent for the treatment of OC, used after carboplatin- and paclitaxel-based chemotherapy [366,367,368,369,370,371,372].

Immunotherapy in OC includes CKIs such as pembrolizumab, nivolumab, ipilimumab, avelumab, atezolizumab, and durvalumab, but response rates remain low (10–15%). Major clinical trials in ovarian cancer have yielded mixed results. The JAVELIN Ovarian 100 trial, evaluating chemotherapy followed by avelumab as maintenance therapy, was discontinued due to lack of clinical benefit, and abagovomab targeting CA-125 also failed to improve outcomes. In contrast, pembrolizumab demonstrated efficacy in metastatic ovarian cancer and was approved by the FDA in 2017 [373].

Therapeutic vaccines targeting TAAs (CA125, p53, FRa, HER2, MAGE-A4, NY-ESO-1) are mostly in early clinical phases. An alternative strategy involves eliminating tumor-associated plasmacytoid dendritic cells (TApDCs), characterized by blood dendritic cell antigen (BDCA-2) expression, as their accumulation correlates with early relapse and reduced OS [374,375,376,377].

Finally, OC cells suppress T-cell activity through CTLA-4, PD-1, LAG-3, and TIM-3. Although monoclonal antibodies targeting these pathways can restore T-cell function, the clinical effectiveness of anti-CTLA-4 and anti-PD-1 therapies in OC remains limited [378].

Another therapeutic option in OC involves targeting the transmembrane immunoregulatory protein B7-H3, which is highly expressed on cancer cells and inhibits NK cell activation as well as promotes pro-inflammatory cytokine release from monocytes/macrophages. By binding CD4^+^ T cells, CD8^+^ T cells, NK and NKT cells, B7-H3 suppresses T-cell proliferation via downregulation of NF-κB. Blocking B7-H3 with monoclonal antibodies increases CD8^+^ T cells and NK cells, contributing to tumor inhibition [379].

CAR-T cell therapy in OC is being developed against MUC-16, mesothelin, and NY-ESO-1, and more recently against Mullerian inhibiting substance type 2 receptor (MISIIR) as MISIIR-CAR-T cells recognize several human OC cell lines in vitro [379,380,381].

Another promising strategy is the use of anti-CCR4 antibodies to inhibit Tregs and deplete CCR4+ T cells. CXCR4 blockade reduces intratumoral Tregs and enhances antitumor immunity, improving survival in murine OC models. Adoptive T-cell therapy is still under evaluation, including neoantigen-specific TCR-engineered T cells, with recent success in identifying novel TCR αβ pairs. NK cell-based therapies are also considered, as NK cells of OC patients show high expression of activating receptors (NKG2D, NKp30, NKp44) following APC stimulation. Additionally, autologous DCs combined with WT-1 peptides, a marker of poor prognosis, are used in OC and are well tolerated [382,383,384,385].

Targeting MDSCs represents another approach, as their blockade enhances immune responses against OC antigens and improves outcomes in high-grade serous tumors. Agents used include metformin, sildenafil, COX-2 inhibitors, nitric oxide (NO) inducers and monoclonal antibodies directed against MDSCs [386].

### 6.12. Prostate Cancer Therapies

Because prostate cancers (PCs) are hormone-driven, most therapeutic strategies target androgens and androgen receptors (ARs). In localized disease, surgery and radiotherapy remain standard, whereas advanced, recurrent, or metastatic cases rely primarily on androgen deprivation therapy (ADT). Despite initial benefit, many patients progress to castration-resistant disease, often through AR pathway reactivation or alternative signaling [387].

**Figure 13:**
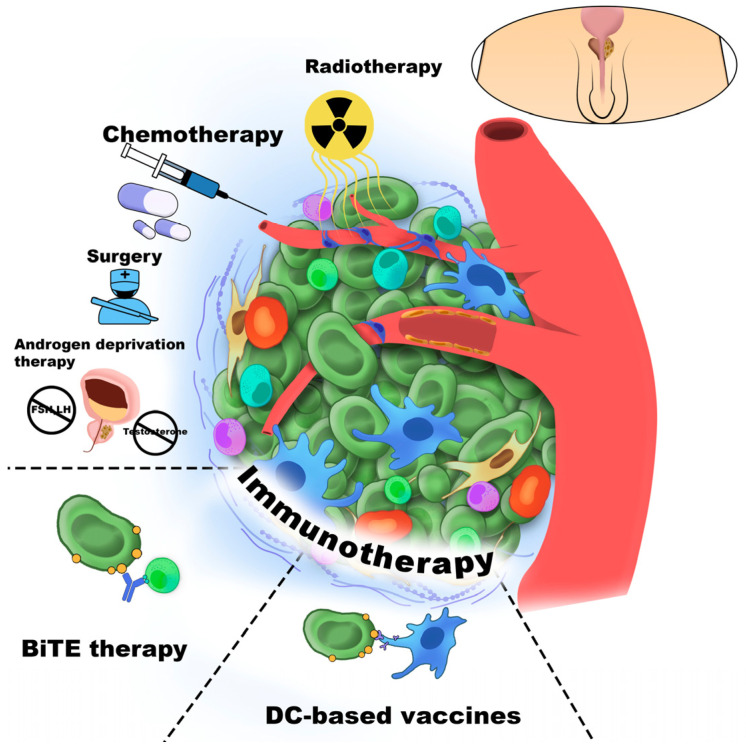
The main therapeutic approaches applied in prostate cancers.

Current treatment options include AR-targeted therapy, radiotherapy, radioligands directed at cell surface proteins or oncogenic kinases, precision therapies targeting DNA repair, CKIs, adoptive cell transfer, chromatin and epigenetic modifiers, antibody–drug conjugates, and bispecific T-cell engagers (BiTEs). The purpose of ADT is to reduce circulating androgens and/or block ARs through surgical castration or GnRH-based hormone therapy, with androgen receptor pathway inhibitors (ARPIs) used as systemic agents [388,389]. Common treatment strategies used in patients with prostate cancers are presented in Figure 13.

In PC, androgens activate CAFs and promote ECM remodeling through the androgen receptor/filamin A complex, enhancing cancer cell migration and invasion. Disrupting this complex with the peptide Rh-2025u reduces pro-tumor ECM changes and inhibits CAF motility. Increased Yes-associated protein 1 (YAP1) expression drives the differentiation of normal fibroblasts into CAFs via the YAP1/TEA domain 1 (TEAD1) complex, altering cytoskeletal protein expression. Inhibiting YAP1 with verteporfin or siYAP1 suppresses CAF proliferation [390,391,392].

Tasquinimod, a quinoline carboxamide small-molecule inhibitor, was evaluated in phase III clinical trials as a TME-targeted therapy. Although it demonstrated anti-angiogenic activity through modulation of hypoxia inducible factor 1 α (HIF-1α0 and VEGF) and promoted macrophage polarization toward the M1 phenotype, it failed to improve median overall survival. Consequently, its development for prostate cancer was discontinued [393].

Immunotherapy for PC includes PD-L1/PD-1 inhibitors, as approximately 8% of primary tumors and up to 32% of metastatic castration-resistant tumors express PD-L1. Pembrolizumab is used in docetaxel-refractory PC. Combining ADT with immunotherapy may enhance therapeutic efficacy; therefore, pembrolizumab has been tested together with enzalutamide (a non-steroidal anti-androgen agent normally administered to patients with castration-resistant prostate cancer) in castration-resistant PC.

Although CTLA-4 and PD-1/PD-L1 inhibitors are considered promising in prostate cancer, monotherapy with nivolumab or pembrolizumab has not improved overall survival in patients with metastatic castration-resistant disease (mCRPC), nor has ipilimumab combined with radiotherapy. In contrast, CTLA-4-targeting antibodies showed clinical benefit in mCRPC patients with high CD8^+^ T-cell infiltration. In metastatic, chemotherapy-naive castration-resistant prostate cancer, ipilimumab increased median PFS and enhanced the prostate-specific antigen response rate compared with placebo. Nevertheless, better clinical results were obtained after a combination of ipilimumab and nivolumab [394,395,396,397].

Another strategy is BiTE therapy. BiTEs are two-headed antibodies that simultaneously bind cancer-associated antigens and T cell markers. In PC, BiTE molecules typically target CD3 on T cells and prostate-specific membrane antigen (PSMA) on prostate cancer cells. Because PSMA is a type II transmembrane protein strongly expressed in metastatic PC, PSMA-directed BiTEs show encouraging activity in mCRPC.

Acapatamab, used as monotherapy or in combination with abiraterone or enzalutamide, is currently being evaluated in clinical trials in patients with mCRPC who are non-responsive to hormonal therapy and taxanes, and has shown improvement in OS [398,399].

Similarly, CAR-T cell therapy can be directed against PSMA. PSMA-CAR-T cells survive, proliferate, and effectively recognize PSMA-positive tumor cells in vivo. Their antitumor activity may be further enhanced by combining them with anti-IL23 monoclonal antibodies. Another relevant CAR-T target is prostate stem cell antigen (PSCA), a glycosylphosphatidylinositol-anchored surface protein highly expressed in bone metastases and increasing with disease progression. CD28-PSCA-CARs have demonstrated antitumor activity in PC models. Additional proposed CAR-T targets include prostate-specific antigen (PSA), prostatic acid phosphatase (PAP), EpCAM, and Transient receptor potential cation channel subfamily M member 8 (Trp-p8), although each carries specific limitations not discussed here [400,401,402,403,404].

Therapies using anti-EpCAM antibodies show promising effects in suppressing PC. Similarly, the use of napabucasin and ruxolitinib, which inhibit the JAK/STAT3 pathway, reduces the viability of prostate CSCs, thereby limiting tumor growth [405].

Among vaccine-based therapies, Sipuleucel-T, a dendritic cell vaccine activating CD4^+^ and CD8^+^ T cells, is approved for mCRPC. In this approach, autologous leukocytes collected by leukapheresis are co-cultured with PSA and other activators to generate mature dendritic cells, which are then reinfused to stimulate T cell responses. In a phase III clinical trial, treatment of patients with metastatic asymptomatic CRPC with sipuleucel-T resulted in a significant improvement in OS [139,406].

Besides DC vaccines, antigen vaccines directed against PSA, PAP, and PSMA are also under development. These vaccines aim to enhance antigen-specific cytotoxic T cell responses and promote selective elimination of prostate cancer cells [407].

Another emerging option includes nucleic acid vaccines. DNA-based vaccines use plasmids encoding PSA, PAP, or PSMA, delivered intramuscularly or subcutaneously to induce immune activation. RNA-based vaccines are considered more effective because they do not integrate into the genome, require only cytoplasmic entry, and can activate even immature dendritic cells, facilitating strong immune stimulation [408,409].

In summary, the effectiveness of vaccines in prostate cancer depends on their combination with cytokines and co-stimulatory molecules that enhance APC activation and modulate T and B cell responses. Key mediators include GM-CSF (APC activation), TNFα (CD8^+^ T cell activation), IL-15 (CD8^+^ T cell activation and memory development), as well as IL-2, IL-12, and IFNγ (immune response modulation) [95].

### 6.13. Renal Cancer Therapies

The choice of treatment in renal cell carcinoma (RCC) depends on disease stage. Localized RCC is typically managed with nephrectomy, although recurrence reaches nearly 20%. Radiotherapy is generally avoided, as RCC is considered radioresistant. Metastatic RCC is treated with immunotherapy and targeted agents, including TKIs [410,411].

Combining immune checkpoint inhibitors with VEGRF-TKIs represents a promising strategy, leveraging interactions between immune and angiogenic pathways. CKIs targeting PD-1/PD-L1 and CTLA-4 (such as pembrolizumab, nivolumab, atezolizumab, and ipilimumab) have demonstrated clinical benefits in advanced RCC, with additional agents targeting VISTA, TIM3, LAG3, and TIGIT currently under evaluation [412,413,414].

Bevacizumab is approved for RCC and, when combined with atezolizumab, enhances Th1-associated gene expression and CD8^+^ T cell infiltration. Conversely, pazopanib reduces CD8^+^ T cell infiltration and increases PD-L1 expression, whereas sunitinib reduces Tregs and MDSCs, improves Th1 responses, and increases overall T cell infiltration [415].

Sunitinib also inhibits Flt-3, CD117 (c-kit), and VEGFR on MDSCs and on cancer cells. Nivolumab is additionally recommended for metastatic RCC beyond first progression [416].

CAR-T cell therapy targeting carbonic anhydrase IX (CAIX) has been tested but with limited success. Other antigens under investigation include 5T4 and CD70, both frequently overexpressed in RCC. CAR-NK cell approaches, such as CD70-specific IL-15-expressing CAR-NK cells, and CAR-Tregs are also being evaluated [34,210,417].

Therapies targeting monocytes, MDSCs, or TAMs include entinostat, which inhibits monocytes and neutrophils, and X4P-001, a CXCR4 inhibitor enhancing T cell-mediated tumor elimination by reducing immunosuppressive MDSCs. These approaches are primarily intended for advanced RCC. Pegilodecakin (pegylated IL-10), combined with anti-PD-1 therapy, increases CD8^+^ T cell numbers but carries risks such as hemophagocytic lymphohistiocytosis [418].

Several vaccine strategies are under investigation in RCC, including HSP complex 96, attenuated modified Ankara virus, DC pulsed with tumor lysates, DC–cancer cell hybrids, and irradiated tumor cells with adjuvants. The IMA901 vaccine, composed of nine HLA class I-binding tumor-associated peptides (TUMAPs), is immunogenic and recommended in combination with GM-CSF to enhance multiclone T cell responses. Combinations of vaccines such as MPDL3280A with avastin or sunitinib are also being explored for advanced or metastatic RCC [419,420].

In a phase III trial in renal cancer patients, vaccination with autologous tumor lysates after nephrectomy improved OS, with particularly favorable outcomes observed for personalized peptide vaccines. The greatest benefit was achieved when peptide-based vaccines were combined with targeted therapies or cytokines [421].

### 6.14. Bladder Cancer Therapies

The choice of management in bladder cancers (BCc) depends on disease stage at diagnosis. Non-muscle invasive tumors are primarily treated with transurethral resection of bladder tumor followed by a single postoperative dose of immunotherapy with BCG or chemotherapy (mitomycin C, epirubicin, or DOX). Patients with resectable, non-metastatic, muscle-invasive BCc are treated with radical cystectomy and bilateral pelvic lymphadenectomy, often combined with cisplatin-based chemotherapy. In metastatic or unresectable disease, systemic chemotherapy remains the main therapeutic option. Cisplatin is recommended for patients with lymph node involvement, while cisplatin plus gemcitabine is used for unresectable, locally advanced, or metastatic transitional cell carcinoma [422,423,424].

In BC, targeting TGF-β is an important CAF-related therapeutic strategy. Selective inhibition of TGFβ1 with the fully humanized monoclonal antibody SRK-181, combined with anti-PD-1 therapy, can overcome primary resistance of cancer cells to CKIs [424].

Immunotherapy is applied at various stages of BC due to elevated PD-1/PD-L1 and CTLA-4 expression. Engineered viral vectors, especially adenoviruses, represent a key treatment avenue. CKIs show clinical benefit in advanced BCc, although their efficacy in high-grade non-muscle invasive bladder cancer (NMIBC) previously treated with BCG remains unclear. Importantly, BCc was the first malignancy treated with BCG, which reduces recurrence and progression by inducing a local immune response following internalization by cancer cells [425,426,427].

The strength of viral-vector therapies lies in their localized activity within the bladder. One approach uses oncolytic adenoviruses encoding human GM-CSF to enhance myeloid cell activation, with possible synergy when combined with pembrolizumab. Another is adstiladrin, a recombinant adenovirus expressing IFNa, supported by the polyamide Syn-3 to improve urothelial entry. Its E2F-1 promoter restricts E1A expression to cells with mutated retinoblastoma pathway, increasing specificity. These therapies show minimal bladder toxicity. Additional strategies include adenoviruses encoding human IFNA2B to enhance NK cell cytotoxicity, although glycosaminoglycans-rich urothelium may affect transduction efficiency [428,429,430].

NK cell-based therapies are also being explored. Immunomodulatory drugs, such as lenalidomide and pomalidomide, enhance NK cell cytotoxicity toward bladder cancer cells. Lenalidomide is well tolerated and may be combined with BCG vaccination to reduce tumor burden. Small-molecule inhibitors of TGFβ type I receptor kinase, vactoserib and galunisertib, represent additional options, particularly for patients who do not respond to PD-1/PD-L1 inhibitors. Because TGFβ enhances BCc aggressiveness, its inhibition may improve NK cell activation [431].

Finally, suppression of stemness and metastatic behavior through inhibition of the Notch2 pathway in CSCs is a promising target in limiting BCc progression [432].

### 6.15. Skin Cancer Therapies

Skin cancer therapies include surgery, radiotherapy, and chemotherapy. However, due to their negative impact on patient well-being, these methods are less preferred. More favorable options include photodynamic therapy (PDT) and photothermal therapy (PTT), which offer high efficacy and safety [433].

**Figure 14:**
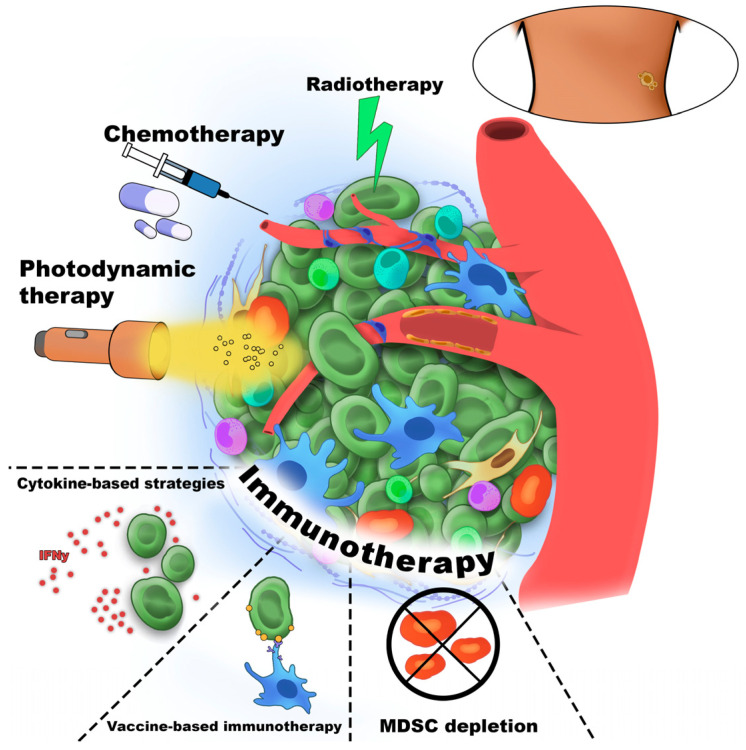
Most commonly used treatment strategies for skin cancers.

In melanoma, photodynamic immunotherapy (PIT)—combining the cytotoxic effects of PDT with stimulation of anti-cancer immunity—is increasingly preferred. PIT acts locally on tumor lesions, reducing systemic toxicity [434]. Common treatment strategies used in patients with skin cancers are presented in Figure 14.

Immunotherapy in melanoma relies heavily on CKIs. Ipilimumab is approved for melanoma treatment, nivolumab is used in metastatic melanoma, and pembrolizumab is applied in advanced cases. Pembrolizumab is also recommended as adjuvant therapy for resected stage III melanoma, improving recurrence-free survival [435,436].

Ipilimumab as monotherapy improves survival in pretreated advanced melanoma. Its combination with sagramostim further enhances outcomes in unresectable stage III–IV melanoma. Currently, the combination of ipilimumab and nivolumab offers superior PFS compared with either agent alone. Similarly, combining ipilimumab with talimogene laherparepvec is effective for advanced unresectable melanoma, despite mild side effects such as fatigue, chills, and diarrhea. Tremelimumab remains an option for refractory or relapsed melanoma, though clinical benefits are modest [39,416,437].

Nivolumab provides favorable outcomes in advanced melanoma, both in wild-type BRAF cases and in patients with BRAF V600 mutations, including >30% reduction in target lesions even beyond progression. In metastatic melanoma, bevacizumab combined with ipilimumab reverses pro-tumor activity of tumor endothelial cells (TECs) by increasing E-selectin, ICAM1, and VCAM1 expression, enhancing T cell recruitment into TME [438,439].

Circulating PD-1+ Tregs may serve as biomarkers of response to pembrolizumab or nivolumab, as their early disappearance correlates with lower risk of progression and metastasis. Beyond CTLA-4 and PD-1/PD-L1, additional immune-modulatory molecules—LAG-3, MUC-1, TIM-3, TIGIT, GITR, and sphingosine kinase—influence T cell function. Notably, sphingosine kinase-1 (SK1) overexpression enhances immunosuppression within the TME. Moreover, reduced immunoglobulin heavy chain gamma expression in B cells, along with elevated IL-10 from Bregs, correlates with poor response to anti-CTLA-4 therapy, although no similar effect is seen with anti-PD-1 treatments [440,441,442].

In melanomas, higher numbers of TILs correlate with favorable outcomes and longer OS. Immunotherapy is primarily used for advanced and metastatic disease. TIL-based therapy, often combined with high-dose IL-2 and/or cyclophosphamide, can induce durable responses, although treatment success depends on factors such as treatment duration, tumor cell doubling time, and susceptibility of tumor cells to lysis. Longer telomeres in TILs and higher CD27/CD28 expression are associated with better responses. Notably, TIL therapy has induced complete and lasting responses in advanced melanoma patients previously resistant to IL-2 or ipilimumab [443].

Cytokine-based strategies, particularly IFN administration, are also used as adjuvant therapy in resected stage III melanoma. Type I IFN upregulates HLA class I on melanoma and immune cells, improving immune recognition. Nonetheless, only a subset of patients, especially those with ulcerated primary tumors, derive meaningful benefit [437].

In melanoma, TCR-T cell therapies targeting antigens such as MART-1, gp100, MAGE-3, and NY-ESO-1 are mainly evaluated in phase I–II trials. While complete responses are rare and partial responses predominate, treatment is often limited by significant toxicities. Notably, TCR-T cells directed against NY-ESO-1 appear the safest, achieving objective response rates of ~47% across clinical trials with minimal severe adverse effects [42].

Effective vaccine-based immunotherapy for melanoma may target melanoma cells directly and use DCs, peptides, or viral vectors. Although DC-based vaccines show limited efficacy, viral vectors such as replication-competent HSV have demonstrated safety in murine melanoma models and human cell lines [444].

DCVs combined with adjuvants that act during the priming phase of the immune response and reduce Treg activation remain under investigation. Activated DCs secrete TNFα, IFNα/β, and IL-12p40/p70, which stimulate CD8^+^ T cells and promote tumor regression. Clinical trials using monocyte-derived DCs pulsed with GM-CSF and IL-4 and directed against MART-1, MAGE-1, MAGE-4, gp100, or tyrosinase peptides induced tumor regression in fewer than half of treated patients [445].

Additional strategies include introducing mRNA encoding CD40L, CD70, and toll-like receptor-4 (TLR4)—TriMix into DCs, or using non-specific adjuvants such as attenuated *Mycobacterium BCG*, *Salmonella*, or *Listeria monocytogenes*. The latter enhances melanoma cell apoptosis at low doses and expands CD8^+^ T-cell cytotoxicity [446].

Biomaterials and nanomaterials may further enhance DC and T-cell activity. PLGA supports DC trafficking and activation, while esomeprazole-coated nanoparticles modulate melanoma cell pH and suppress immunosuppressive myeloid cells, thereby activating T cells and NK cells. In murine models, PLGA combined with GM-CSF, CpG, or tumor lysates shows greater cytotoxicity than PLGA with esomeprazole alone. Unmodified DCs may also be used in melanoma therapy, as they effectively generate cytotoxic T cells and can be isolated directly from blood. Autologous DC-based immunotherapy has shown results superior to dacarbazine in melanoma patients [447,448,449,450].

The strategy of targeting MDSCs in melanoma includes five main approaches: depleting MDSCs, inhibiting their suppressive activity, blocking their recruitment and expansion, promoting their differentiation, and inhibiting their metabolism. Agents such as gemcitabine, 5-FU, PTX, and DOX effectively reduce MDSCs in both mouse melanoma models and human patients. Additional options include gemtuzumab ozogamicin (anti-CD33 monoclonal antibody) and sunitinib, which further decrease MDSCs counts. PI3K inhibitors, such as IPI-549 combined with nivolumab, enhance MDSC reprogramming into more immune-responsive phenotypes in advanced melanoma resistant to anti-PD-L1 therapy. IDO inhibitors (epacadostat, navoximod, BMS-986205) combined with CKIs also show potential in unresectable or metastatic melanoma [451,452].

Recruitment-blocking strategies involve interfering with GM-CSF/G-CSF signaling, including the use of CSF-1R inhibitors (PD-0360324). Anti-CXCR2 therapy targeting polymorphonuclear myeloid-derived suppressor cells (PMN-MDSCs) in the TME shows promising effects in metastatic melanoma, and the combination of pembrolizumab with SX-682 (CXCR1/2 inhibitor) is being clinically evaluated. In metastatic uveal melanoma, histone deacetylase inhibitors such as entinostat with pembrolizumab or trichostatin A combined with anti-PD-L1 antibodies are considered. Additional approaches include targeting angiogenesis-related MDSC activity using doxycycline, anti-VEGF/VEGFR inhibitors, or tyrosine kinase with immunoglobulin and epidermal growth factor homology domains 2 (TIE-2). Promoting MDSC maturation with ATRA combined with pembrolizumab has shown low toxicity and encouraging efficacy in advanced melanoma. Finally, inhibition of MDSC metabolism using FATP2 inhibitors, etomoxir (fatty acid oxidation inhibitor), or itaconate-pathway inhibitors (linked to immune-responsive gene 1 (IRG1) activation) is being explored to improve patient survival [452,453,454].

## 7. Conclusions

Cancer treatment outcomes depend not only on the intrinsic properties of the cancer cells but also on the composition, functional state, and plasticity of the surrounding microenvironment, which can support or limit the efficacy of anti-cancer therapies.

Modern oncology offers a wide spectrum of therapeutic strategies, including radiotherapy, chemotherapy, targeted therapies, and immunotherapy. However, despite significant therapeutic advances, several substantial challenges remain. Tumor heterogeneity, therapy-induced adaptive resistance, immunosuppressive signaling, and metabolic reprogramming, not to mention physical stromal barriers within the TME, continue to limit clinical response. Furthermore, the functional heterogeneity of TME components across tumor types and disease stages complicates the translation of promising preclinical strategies into broadly effective clinical applications. A particularly instructive example of the complexity of TME-targeted interventions is the paradox of cancer-associated fibroblast targeting. Although CAF-directed therapies have shown efficacy in preclinical models, their clinical translation has been disappointing. This discrepancy reflects the heterogeneity and functional plasticity of CAF populations, which can exert both tumor-promoting and tumor-restraining effects depending on tumor context and disease stage. Importantly, indiscriminate CAF depletion may exacerbate immunosuppression, promote tumor aggressiveness, or increase therapeutic resistance [455]. Thus, future research should focus on integrative approaches that combine molecular, cellular, and spatial profiling of the TME to identify predictive biomarkers of treatment response and resistance. Approaches combining multiple therapeutic strategies aimed at achieving synergistic effects, reducing the risk of resistance, relapse, and systemic toxicity, should become a central paradigm. Immunotherapy with stromal targeting, metabolic modulation, adoptive cell therapies, or oncolytic therapies represent a particularly promising avenue. In parallel, emerging technologies, including multiomics platforms, artificial intelligence-assisted data integration, and synthetic biology approaches, are expected to support the rational design of next-generation therapeutic strategies, including nanovaccines and other advanced delivery systems, while improving target specificity and treatment personalization.

Importantly, future therapeutic development should balance innovation with feasibility, prioritizing approaches that are cost-effective and compatible with clinical translation. Standardized data sharing and well-designed clinical trials incorporating appropriate patient selection criteria will be essential to accelerate progress. In summary, effective cancer treatment requires not only accurate diagnosis and classification but also a thorough understanding of interactions of the tumor with its microenvironment and the individualized application of therapeutic options. Continued efforts to uncover and exploit these interactions, supported by emerging technologies and interdisciplinary collaboration, will be crucial to the development of next-generation cancer therapies that not only eradicate cancer cells but also reprogram the tumor microenvironment towards sustained antitumor immunity.

## Figures and Tables

**Table 1:** Classification of the main groups of immune checkpoint inhibitors according to their primary targets.

Type of CKIs	Key Target	Mechanism of Action	Representative Drugs
PD-1 inhibitors	T cells	Blockade of PD-1 signaling on T cells, restoring antitumor immune activity	Nivolumab Pembrolizumab Cemiplimab Dostalimab
PDL-1 inhibitors	Cancer cells	Blockade of the PD-L1 ligand binding with PD-1 receptor expressed on T cells	Atezolizumab Avelumab Durvalumab
CTLA-4 inhibitors	T cells	Blockade of the CTLA-4 protein expression on T cells	Ipilimumab Tremelimumab
LAG-3 inhibitors	T cells	Inhibition of LAG-3-MHC interaction on T cells	Relatlimab Eftilagimoid alpha TSR-033 Bavunalimab
TIM-3/HAVCR2 inhibitors	T cells, NK cells	Blockade of the inhibitory signals from TIM-3 receptor	Sabatolimab Cobolimab
B7-H3 inhibitor	T cells, NK cells	Inhibition of B7-H3-mediated suppression of T cell activation	Enoblituzumab Ifinatamab Deruxtecan MGC018 MGA271
TIGIT inhibitor	T cells, NK cells	Inhibition of the TIGIT–ligand interactions	Tiragolumab Etigilimab Ociperlimab
CD47-SIRPα	macrophages	Disruption of the CD47-SIRPα interaction between cancer cells and macrophages	Magrolimab Lemzoparlimab Letaplimab Evorpacept IMM-0306 RRx-001
NKG2A inhibitors	T cells, NK cells	Inhibition of NKG2A to prevent interaction with HLA-E overexpressed on cancer cells	Monalizumab BRY805
VISTA inhibitors	T cells, myeloid cells	Reversal of T cell inhibition and modulation of the TME, enhanced cytokine production	SNS-101 HMBD-002 PMC-309 KVA12123 CA-170 MG-V-53 Chidamide
PVRIG/PVRL2 inhibitors	T cells, NK cells	Inhibition of the interaction between immune cells and cancer cells	COM701
ADORA2A inhibitors	immune cells	Disruption of adenosine A2A receptor-mediated immune suppression	Caffeine ZM241385 JNJ-41501798
BTLA2 inhibitors	B cells, T cells	Prevention of inhibitory B and T cell attenuator receptor-ligand binding	HVEM(14–39) 6A6
TACTILE inhibitors	NK cells, T cells,	Blockade of the inhibitory receptor CD96 on immune cells	under investigation
SIGLEC-15 inhibitors	TAMs	Disruption of the T cells suppression	NC318 1-15D1

**Table 2:** Characteristics of viruses used in oncolytic virus cancer therapy.

Virus	Genome	Methods of Entry	Immunogenic Potential	Advantages	Disadvantages
Herpes virus	dsDNA	membrane penetration and fusion	low	large genome susceptible to genetic manipulation, replicates only within cells	high pathogenicity
Vaccinia virus	dsDNA	macropinocytosis	high	rapid and efficient spreading, good insertion capacity	pathogenicity
Coksackie virus	ssRNA	endocytosis	high	high potential to induce anti-cancer immune response, low toxicity	detailed strain selection requirement, high risk of systemic spread
Adenovirus	dsDNA	endocytosis	low	produces high concentration of viral particles easily available susceptible to genetic manipulation, displays strong lytic activity easily combined with other immunotherapies	tropism for majority of tissues attenuated viral spreading
Reovirus	dsRNA	endocytosis	low	possible intravenous administration low risk of toxicity	low susceptibility to gene editing
Measles virus	ssRNA	membrane fusion	low	susceptible to genetic manipulation	pathogenicity
Newcastle disease virus	ssRNA	endocytosis, pH-independent fusion	low	low immunogenicity in humans, susceptible to multicentric replication, rapidly spreading	low susceptibility to gene editing systemic toxicity
Sindbis virus	ssRNA	receptor-mediated endocytosis	high	causes strong anti-cancer immune response high specificity for cancer cells	potential cytotoxicity short-term activity

**Table 3:** Classification of cancer vaccines regarding the mechanism of action.

Types	Vaccine Composition	Results of Vaccination
Peptide-based vaccines	Short synthetic amino acids sequences (20–30) derived from tumor antigens, often combined with adjuvants	Activation of DCs, macrophages, B cells, CTLs and Th cells
Nucleic acid-based vaccines	DNA plasmids or mRNA (non-replicating, unmodified, modified or self-amplifying mRNA isolated from viruses)	Activation of DCs, macrophages, CTLs and Th cells
Cell-based vaccines	Allogeneic or autologous DCs combined with tumor antigens, whole/lysed cancer cells combined with tumor antigens	Activation of DCs, macrophages, CTLs, Th and NK cells
IPSc-based vaccines	Reprogrammed patient-derived iPSCs used as whole cells, cell lysates or genetically modified derivatives combined with adjuvants	Activation of DCs, macrophages, NK cells, CTLs and Th cells
In situ cancer vaccines	Patient-derived tumor cells combined with immune stimulating factors (cytokines, peptides, neoadjuvants)	Activation of DCs, macrophages, neutrophils, NK cells, CTLs and Th cells
Viral/bacterial vector-based vaccines	Genetically modified viruses with TAAs or bacterial elements (flagellin), or genetically modified bacteria combined with TAAs	Activation of DCs, macrophages, NK cells, CTLs and Th cells
Exosome-based vaccine	MHC class I and II, HSPs proteins, TAAs, miRNAs	Activation of DCs, CTLs and Th cells

## Data Availability

No new data were created or analyzed in this study.

## Abbreviations

The following abbreviations are used in this manuscript:

A2R	adenosine A2 receptor
ACT	adoptive cell therapy
ADCC	antibody-dependent cell-mediated cytotoxicity
AdHu5	adenovirus human serotype 5
ADORA2A	adenosine A2A receptor/A2aR
ADT	androgen deprivation therapy
AdV	adenovirus
AFP	alpha-fetoprotein
AFP-pulsed DCs	alpha-fetoprotein peptide-pulsed autologous dendritic cells
ALDH	aldehyde dehydrogenase
ALL	acute lymphoblastic leukemia
AMPK	adenosine monophosphate-activated protein kinase
APCs	antigen-presenting cells
APOL3	apolipoprotein
AR	androgen receptor
Aregs	adipogenesis regulatory cells
ARG	arginase
ARPIs	androgen receptor pathway inhibitors
aSMA	alpha anti smooth antibody
ATC	anaplastic thyroid cancer
ATLs	autologous tumor lysates
ATP	adenosine triphosphate
ATRA	all-trans retinoic acid
AuTLs	autologous tumor-stimulated cytotoxic T cells
AXL	AXL receptor tyrosine kinase
B7-H3	B7 homolog 3 protein
BAT3	Bcl-2 antagonist of cell death 3
BC	breast cancer
BCc	bladder cancer
BCG	Bacillus Calmette–Guerin
BCMA	B-cell maturation antigen
BDCA-2	blood dendritic cell antigen
BiKEs	bifunctional killer engagers
BiTEs	bispecific T-cell engager
BMI1	B cell-specific Moloney murine leukemia virus integration site 1
BMSCs	bone-marrow stem cells
BRAF	B-Raf proto-oncogene
BRCA	breast cancer gene
Bregs	B regulatory cells
BTK	Bruton tyrosine kinase
CAFs	cancer-associated fibroblast
CAIX	carbonic anhydrase IX
CAP	cold atmospheric plasma
CAR	chimeric antigen receptor
CAR-M	chimeric antigen receptor macrophages
CAR-P	chimeric antigen receptor-phagocytes
CCL-2	chemokine ligand
CCR2	C-C chemokine receptor type 2
CDX1	caudal type homobox 1
CEA	carcinoembryonic antigen
CEACAM-1	carcinoembryonic antigen-related cell adhesion molecule-1
CKIs	immune checkpoint inhibitors
CLL	chronic lymphocytic leukemia
COX-2	cyclooxygenase-2
CP	cyclophosphamide
CRC	colorectal cancer
CRS	cytokine release syndrome
CSCs	cancer stem cells
CSFR-1	colony stimulating factor receptor 1
CTA	cancer testis antigen
CTCL	cutaneous T-cell lymphoma
CTL	cytotoxic T cells
CTLA-4	cytotoxic T-lymphocyte-associated protein 4
CXCR4	CXC motif chemokine receptor 4
DAMPs	damage-associated molecular patterns
DART	Dual affinity retargeting technology
DCs	dendritic cells
DCVs	dendritic cells-based vaccines
DFS	disease-free survival
dMMR	mismatch repair deficient
DOX	doxorubicin
E1A	early gene product
EBV	Epstein–Barr virus
EC	esophageal cancer
ECM	extracellular matrix
EGCG	epigallocatechin-3-gallate
EGFR	epithelial growth factor receptor
eIF2	eukaryotic initiation factor 2
EMT	epithelial–mesenchymal transition
EpCAM	epithelial cell adhesion molecule
EphA2	ephrin type-A receptor 2
ER	estrogen receptor
Erk	extracellular signal-regulated kinase
ES-SCLC	extensive-stage small lung cancer
EVs	extracellular vesicles
FasL	Fas ligand
FAP	fibroblast activation protein
FAP	transmembrane serine protease
FATP2	fatty acid oxidation inhibitor
FDA	Food and Drug Administration
FGFR-1	fibroblast growth factor receptor 1
FL	follicular lymphoma
FLT3	FMS-like tyrosine kinase 3
FMOD	fibromodulin
FoxP3	forkhead box P3
FRa	folate receptor alpha
FTC	follicular thyroid carcinoma
5-FU	5-florouracil
GAAs	glioma-associated antigens
Gal-9	galectin-9
GBP2	guanylate-binding protein 2
GC	gastric cancer
GCC	guanylyl cyclase C
GD2	disialoganglioside
GITR	glucocortycoid-induced tumor necrosis factor
GLS1	glutaminase 1
GM-CSF	granulocyte-macrophage stem cell factor
GTP	guanosine triphosphate
GvHD	graft-versus-host disease
HA	hyaluronic acid
HAVCR-2	hepatitis A virus cellular receptor 2
HBV	hepatitis B
HCC	hepatocellular carcinoma
HDAC6	histone deacetylase 6
HEV	high endothelial venules
HER-2	human epidermal growth factor receptor-2
HGF	hepatocyte growth factor
HIF1α	hypoxia inducible factor 1 alpha
HIPEC	hyperthermic intraperitoneal chemotherapy
HLA	human leukocyte antigen
HMGB1	high mobility group box 1
HNC	head and neck cancer
HPV	human papilloma virus
HR+	hormone receptor-positive
HRV2	human rhinovirus type 2
HSCs	hepatic stellate cells
HSP	heat shock protein
HSV	herpes simplex virus
hTERT	human telomerase reverse transcriptase
ICAM-1	intracellular adhesion molecule 1
ICANS	immune effector cell-associated neurotoxicity synfrome
ICD	immunogenic cell death
ICOS	inducible T cell co-stimulatory factor
ICP	infected cell protein
IDH1	isocitrate dehydrogenase
IDO-1	indoleamine-2,3-dioxygenase 1
IFNγ	interferon gamma
IL	interleukin
IL-R	interleukin receptor
IRG-1	immune-responsive gene-1
FcRγ	IgY-Fc receptor
IgSF	immunoglobulin superfamily
IgV	immunoglobulin variable domain
iPSCs	induced pluripotent stem cells
IRES	internal ribosome entry site
KIRs	killer-immunoglobulin like receptors
KLF	Krupple-like factor
KRAS	Kirsten rat sarcoma viral oncogene
LAG-3	lymphocyte activation gene 3
LAT	latency-associated transcript
LBCL	large B-cell lymphoma
LC	liver cancer
LDHA	lactate dehydrogenase A
LH-RH	luteinizing hormone-releasing hormone
LPrA2	leptin peptide receptor antagonist 2
LRRC15	leucine-rich repeat-containing protein 15
Ly6	leukocyte antigen
MAGE	melanoma-associated antigen
MAP7D2	microtubule-associated protein
MAPK	mitogen-activated protein kinase
MART-1	melanoma antigen recognized by T cells 1
MCL	mantle cell lymphoma
MCP-1	macrophage chemoattractant protein-1
mCRC	metastatic colorectal cancer
mCRPC	metastatic castrate resistant prostate cancer
MDSCs	myeloid-derived suppressor cells
MEK	mitogen-activated protein kinase
MGMT	O6-methylguanine-DNA-methyltransferase
MISIIR	Mullerian inhibiting substance type 2 receptor
MHC	major histocompatibility complex
MM	myeloma multiplex
MMP	matrix metalloproteinase
dMMR	Deficient mismatch repair
MSCs	mesenchymal stem cells
MSI	microsatellite instability
MSI–(H)	microsatellite instability (high)
MSLN	mesothelin
MSS	microsatellite stable
MTC	medullary thyroid cancer
MV	measles virus
MV-EZ	MV-Edm-Zagreb
MVs	microvesicles
MUC-1	mucin-1
myCAFs	myofibroblastic cancer-associated fibroblasts
MZL	marginal zone lymphoma
NDV	Newcastle disease virus
NECL	nectin-like
NFκB	nuclear factor kappa-light-chain enhancer of activated B cells
NIS	sodium iodide symporter
NK	natural killer cells
NKG2A	natural killer group 2 member A
NKp30	natural cytotoxicity triggering receptor 3
NMIBC	non-muscle invasive bladder cancer
NO	nitric oxide
Notch-1	neurogenic locus notch homolog protein 1
NRAS	neuroblastoma rat sarcoma virus viral oncogene homolog
NSCLC	non-small cell lung cancer
NY-ESO-1	New York esophageal squamous cell carcinoma 1
OC	ovarian cancer
OMVs	outer membrane vesicles
ORR	overall response rate
OS	overall survival
OSCC	oral squamous cell carcinoma
OV	oncolytic viruses
PAI-1	plasminogen activator inhibitor-1
PAP	prostatic acid phosphatase
PARP	poly(ADP-ribose)
PARPi	PARP inhibitor
ParvOryx	parvovirus H1
PBMCs	peripheral blood mononuclear cells
PC	prostate cancer
PCa	pancreatic cancer
pDCs	plasmocytoid dendritic cells
PD-L1	programmed death ligand-1
PDGF	platelet-derived growth factor
PDGFR	platelet-derived growth factor receptor
PDT	photodynamic therapy
PEGPH20	pegvorhyaluronidase alpha
PEG-LPrA2	leptin peptide receptor antagonist 2
PET	positron emission tomography
PFS	progression-free survival
PFVSRIPO	picornavirus-based oncolytic virus
PGE	prostaglandin E
PI3K/mTOR	phosphatidylinositol 3-kinase/mammalian target of rapamycin
PIT	photodynamic immunotherapy
PKR	protein kinase R
PLD	pegylated liposomal doxorubicin
PLGA	poly(lactic-co-glycolic acid)
PMN-MDSCs	polymorphonuclear myeloid-derived suppressor cells
PSA	prostate-specific antigen
PSCA	prostate stem cell antigen
PSCs	pancreatic stem cells
PSMA	prostate-specific membrane antigen
PTC	papillary thyroid carcinoma
PtdSer	phosphatidylserine
PTEN	phosphatase and tensin homolog on chromosome 10
PTT	photothermal therapy
PTX	paclitaxel
PVRIG/PVRL2	poliovirus receptor-related immunoglobulin domain-containing
PVR-like	poliovirus receptor-like
RAR	retinoic acid receptor
RAS	rat sarcoma virus
RCC	renal cell carcinoma
RNS	reactive nitrogen species
ROS	reactive oxygen species
RUC-GFP	Renilla luciferase-Aequorea green fluorescent protein
SCC	squamous cell carcinoma
scFv	single-chain variable fragment
SCLC	small-cell lung cancer
SDF-1	stromal-derived factor-1
SIGLEC	sialic acid-binding immunoglobulinlike lectin
siRNA	small interfering RNA
SIRPα	signal regulatory protein receptor
SK-1	sphingosine kinase 1
SLRP	small leucine-rich repeat proteoglycan
STAT	signal transducer and activator of transcription
STING	stimulator of interferon gene
synNotch	cancer-specific synthetic Notch receptor
TAAs	tumor-associated antigens
TACE	transarterial chemoembolization
TAFs	tumor-associated fibroblasts
TA-HEVs	tumor-associated high endothelial venules
TAMs	tumor-associated macrophages
TANs	tumor-associated neutrophils
TApDCs	tumor-associated plasmocytoid dendritic cells
Tc	T cytototic cell
TCA	Tricarboxylic acid cycle
TCR	T cell receptor
TEAD	TEA domain
TECs	tumor endothelial cells
TGFβ	tumor growth factor
Th	T helper cells
TIBs	tumor-infiltrating B cells
TIE-2	tyrosine kinase with immunoglobulin and epidermal growth factor homology domains 2
TIGIT	T cell immunoreceptor with Ig and ITIM domains
TILs	tumor-infiltrating lymphocytes
TIM-3	T cell immunoglobulin and mucin-domain containing 3
TKIs	tyrosine kinase inhibitors
TLR	toll-like receptor
TLS	T lymphocyte structure
TMB	tumor mutation burden biomarker
TME	tumor microenvironment
TNBC	triple-negative breast cancer
TNFα	tumor necrosis factor alpha
TNFR	Tumor necrosis factor receptor
TNM	tumor, nodes, metastasis
TOE	tumor organismal environment
Tregs	regulatory T cells
TRIB3	tribbles homolog 3
TRIM	T-cell receptor-interacting molecule
Trp-p8	transient receptor potential cation channel subfamily M member 8
TSAs	tumor-specific antigens
Tscm	T cells stem cells
TSH	thyroid-stimulating hormone
TSP-1	thrombospondin-1
TTK	threonine tyrosine kinase
TUMAPs	tumor-associated peptides
UCB	umbilical cord blood
USP	ubiquitin-specific processing protease
VCAM-1	vascular cell adhesion molecule-1
VDR	vitamin D receptor
VEGFR2	vascular endothelial growth factor receptor 2
VHH	single-domain antibodies
VISTA	V-domain immunoglobulin suppressor of T cell activation
VV	vaccinia virus
WT-1	Wilm’s tumor 1
YAP-1	Yes-associated protein 1
ZAP	zinc finger antiviral protein
